# Sapropterin (BH4) Aggravates Autoimmune Encephalomyelitis in Mice

**DOI:** 10.1007/s13311-021-01043-4

**Published:** 2021-04-12

**Authors:** Katja Schmitz, Sandra Trautmann, Lisa Hahnefeld, Caroline Fischer, Yannick Schreiber, Annett Wilken-Schmitz, Robert Gurke, Robert Brunkhorst, Ernst R. Werner, Katrin Watschinger, Sabine Wicker, Dominique Thomas, Gerd Geisslinger, Irmgard Tegeder

**Affiliations:** 1 Institute of Clinical Pharmacology, Medical Faculty, Goethe-University, Frankfurt, Germany; 2 Department of Clinical Neurology, Medical Faculty, Goethe-University, Frankfurt, Germany; 3 Institute of Biological Chemistry, Medical University of Innsbruck, Biocenter, Austria; 4 Occupational Health Services, Medical Faculty, Goethe-University, Frankfurt, Germany; 5 Fraunhofer Institute for Translational Medicine and Pharmacology (ITMP), Frankfurt, Germany; 6 Fraunhofer Cluster of Excellence for Immune Mediated Diseases, Frankfurt, Germany

**Keywords:** Tetrahydrobiopterin, T-cells, GTP cyclohydrolase, Nitric oxide, Ceramides, Omega lipids

## Abstract

Depletion of the enzyme cofactor, tetrahydrobiopterin (BH4), in T-cells was shown to prevent their proliferation upon receptor stimulation in models of allergic inflammation in mice, suggesting that BH4 drives autoimmunity. Hence, the clinically available BH4 drug (sapropterin) might increase the risk of autoimmune diseases. The present study assessed the implications for multiple sclerosis (MS) as an exemplary CNS autoimmune disease. Plasma levels of biopterin were persistently low in MS patients and tended to be lower with high Expanded Disability Status Scale (EDSS). Instead, the bypass product, neopterin, was increased. The deregulation suggested that BH4 replenishment might further drive the immune response or beneficially restore the BH4 balances. To answer this question, mice were treated with sapropterin in immunization-evoked autoimmune encephalomyelitis (EAE), a model of multiple sclerosis. Sapropterin-treated mice had higher EAE disease scores associated with higher numbers of T-cells infiltrating the spinal cord, but normal T-cell subpopulations in spleen and blood. Mechanistically, sapropterin treatment was associated with increased plasma levels of long-chain ceramides and low levels of the poly-unsaturated fatty acid, linolenic acid (FA18:3). These lipid changes are known to contribute to disruptions of the blood–brain barrier in EAE mice. Indeed, RNA data analyses revealed upregulations of genes involved in ceramide synthesis in brain endothelial cells of EAE mice (LASS6/CERS6, LASS3/CERS3, UGCG, ELOVL6, and ELOVL4). The results support the view that BH4 fortifies autoimmune CNS disease, mechanistically involving lipid deregulations that are known to contribute to the EAE pathology.

**Supplementary Information:**

The online version contains supplementary material available at 10.1007/s13311-021-01043-4.

## Introduction

GTP cyclohydrolase, GCH1, is the rate-limiting enzyme in the de novo biosynthesis of tetrahydrobiopterin (BH4), which is an enzyme cofactor essentially required for the production of monoamine neurotransmitters and nitric oxide [1], and the metabolism of ether-lipids via alkylglycerol monooxygenase (AGMO) [2]. The synthesis is a 3-step enzymatic cascade starting with GCH1. The downstream enzymes are PTPS (pyruvoyltetrahydropterin synthase) and SPR (sepiapterin reductase) (pathway in Suppl. Fig. 1). The expression of GCH1 is increased on demand to meet requirements of BH4, which is high in inflammatory conditions owing to the upregulation of inducible nitric oxide synthase in myeloid-derived inflammatory cells [3].

**Fig. 1:**
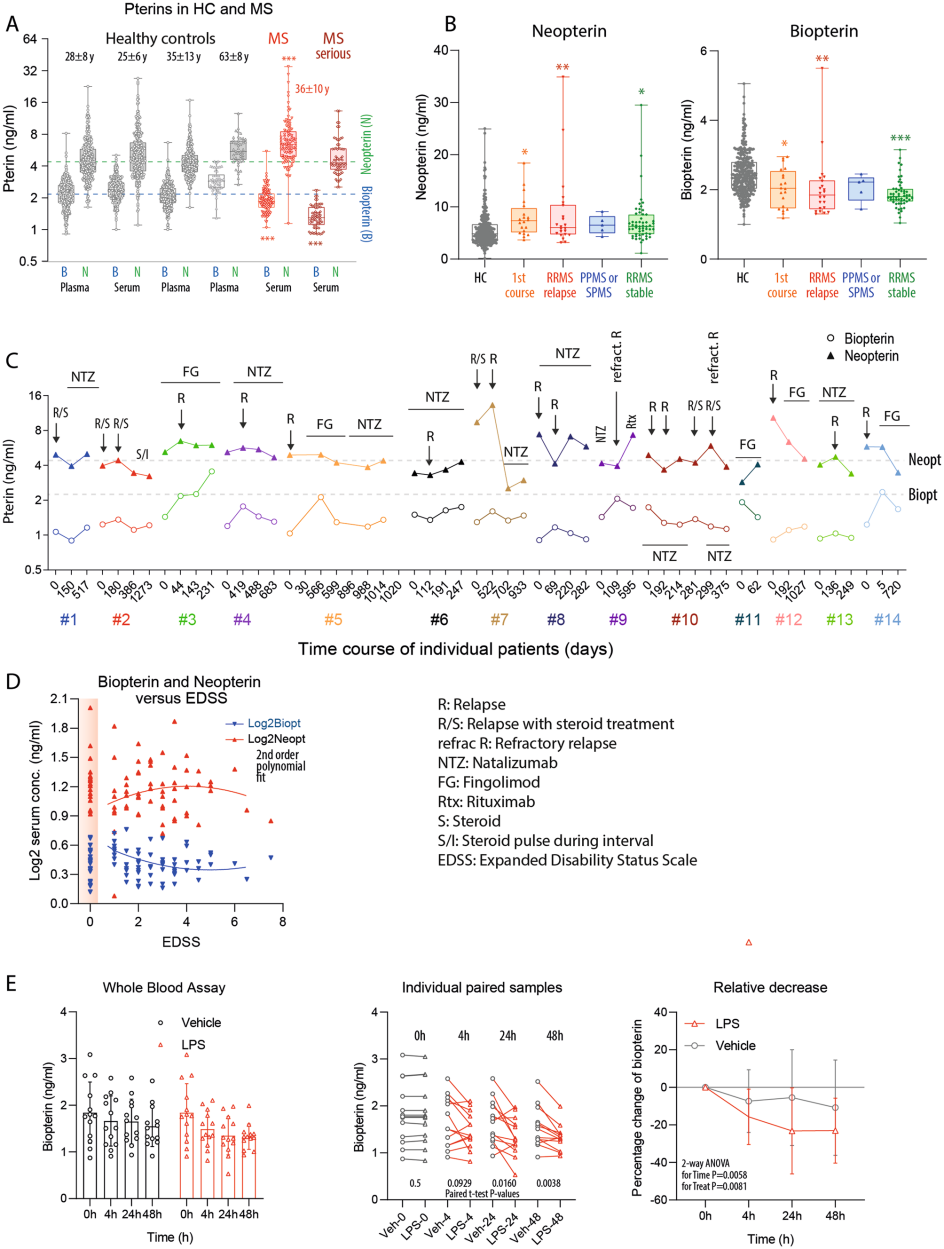
Biopterin and neopterin in human multiple sclerosis.** a** Biopterin and neopterin concentrations in plasma or serum in two cohorts of patients with multiple sclerosis (MS-1, *n* = 102; MS-2, *n* = 14, with repeated samples over time) as compared to biopterin/neopterin in four cohorts of healthy control subjects of different ages. The gender distribution was 2:1 women/men in MS patients as well as in healthy cohorts. The time courses are shown in **c**. The box shows the interquartile range, the line is the median, whiskers show minimum to maximum. Each scatter represents one subject except in the right plot, where each scatter is one sample. Data were compared with two-way ANOVA for “pterin × group” and subsequent post hoc *t* test for group using an adjustment of alpha according to and Šidák. *P < 0.05, **P < 0.01, ***P < 0.001. **b** Serum biopterin concentrations of MS cohort-1 categorized according to ICD10 criteria as compared to healthy controls. Each scatter is a patient or healthy control. The box shows the interquartile range, the line is the median, whiskers show minimum to maximum. Data were compared with two-way ANOVA for “pterin × group” and subsequent post hoc *t* test for group using an adjustment of alpha according to and Šidák. *P < 0.05, **P < 0.01, ***P < 0.001. **c** Time courses of serum biopterin and neopterin concentrations in 14 MS patients with complicated MS course. The *X*-axis shows the days since diagnosis, which was day 0. The dashed lines show the mean biopterin (B) or neopterin (N) concentration in healthy controls. Arrows point to relapses (R) without or with steroid medication (R/S). Patients received fingolimod (FG), natalizumab (Ntx) or rituximab (Rtx) as indicated. Biopterin was persistently low, not obviously in association with relapse or medication. **d** Association of biopterin and neopterin serum concentrations with the EDSS score at the time of taking the serum sample. A second order binomial fit was used to describe the association. Patients with EDSS of “zero” or unknown EDSS were not included in the fit, and they are shown in the left with red background. **e** Plasma concentrations of biopterin in a human whole blood assay upon stimulation with lipopolysaccharide, LPS versus vehicle (mean, SD). Biopterin levels drop over time in LPS stimulated samples. Each scatter is one healthy donor (*n* = 13), whose blood samples were split in two, one for LPS the other as control. The middle panel shows the paired analyses of the subject’s LPS and vehicle samples at different time points. Paired data were compared by paired t-tests and time courses per 2-way ANOVA for “time × treatment”.

Recently, Cronin et al. demonstrated that GCH1 is also upregulated in activated CD4+ and CD8+ T-cells [4]. By using T-cell-specific GCH1 depletion and overexpression, or SPR inhibition, the authors show that BH4 acts as a regulator of T-cell receptor dependent T-cell proliferation in various models of allergy, autoimmunity, and immune mediated cancer surveillance [4]. GCH1 deficiency attenuated T-cell proliferation, and overexpression had opposite effects. Intriguingly, nitric oxide (NO) was apparently not involved but BH4 acted as a regulator of iron homeostasis [4], suggested by low iron levels and low conversion of ferri Fe^3+^ to ferro Fe^2+^. As a result, cytochrome c activity and ATP generation were reduced (Suppl. Fig. 1). Hence, the proliferation defect might originate from a deficit of ATP [4].

The authors mostly used T-cell transfer models that skip the active immunization. T-cell transfer models do not require antigen recognition, presentation and initiation of antigen-specific T-cell proliferation. The model is important in the light of the protective antioxidative and anti-inflammatory effects of BH4/sapropterin in models of cardiovascular diseases [5, 6] or colitis [7], and inhibition of tumor growth upon GCH1 inhibition [8]. Sapropterin-hydrocholoride (Kuvan®) is a clinically available BH4 drug approved for treatment of genetic BH4 deficiency [9, 10] and was also suggested as adjunctive treatment for cardiovascular disease [11, 12], diabetes [13], depression and schizophrenia [14], and mycobacterial infection [15].

The duality of results of previous studies suggest that the net outcome of high or low BH4 concentrations *in vivo* depends on the cellular source and the complex functions in the disease-specific and site-specific (auto)-immune context. Because sapropterin is an approved drug, it is crucial to know if it boosts T-cell responses under certain conditions. In particular, autoimmune diseases of the peripheral and central nervous system such as multiple sclerosis differ from other sites because blood-to-brain (BBB) and brain-to-CSF barriers normally hinder immune cells from invasion. BH4-dependent endothelial NOS is highly expressed in brain endothelial cells, and pro-oxidative metabolites that are generated via NOS in the absence of BH4 promote a disruption of the BBB [16]. It is well known that MS pathophysiology has a strong oxidative contribution [17–20]. In addition to redox targets, BH4 alters bioactive lipids [7] presumably via AGMO, which are crucial for BBB integrity [21–23].

To assess the putative benefit or adverse effects of the currently available BH4 drug, sapropterin/Kuvan®, we analyzed biopterin and neopterin in patients at various stages of MS, and we used the experimental autoimmune encephalomyelitis (EAE) model with/without sapropterin treatment to assess effects of the drug on the course of the disease. We analyzed disease scores, immune cell proliferation and invasion and alterations of lipid signaling molecules. The mechanistic focus on bioactive lipids was motivated by the functions of AGMO in inflammation and resolution [24], and iron dependency of fatty acid metabolism.

## Results

### Persistently Low Biopterin and High Neopterin Plasma Levels in Multiple Sclerosis Patients

Neopterin is used as activity and prognostic marker in inflammatory diseases, cancer, some infections, and rheumatoid arthritis [25–28]. It is produced in excess if the expression and/or activity of GCH1 exceeds the capacity of the two downstream enzymes (PTPS and SPR) to convert the GCH1 product, neopterin-3-phosphate (neopterin-3P) into BH4 (Pathway in Suppl. Fig. 1). Excess neopterin in serum or plasma mainly originates from activated immune cells and endothelial cells. Overall, MS patients had increased neopterin levels (Fig. 1a, b), but there was no clear association with the disease course in individual patients (Fig. 1c). In parallel, biopterin levels were persistently reduced (Fig. 1a–c). In some patients, there was an inverse association of high neopterin with low biopterin (Fig. 1c), and this was reflected in binomial fits of EDSS (Expanded Disability Status Scale) scores versus biopterin or neopterin (Fig. 1d).

High neopterin but low biopterin suggested excessive consumption of BH4 presumably in activated immune cells. In support, biopterin levels dropped over time in human whole blood assays stimulated with LPS (Fig. 1e). The whole blood assay reveals BH4 turnover, that is not compensated by upregulations of GCH1, which occur upon LPS stimulation of immunocytes in culture [29].

The pterin data of MS patients suggested two alternative interpretations assuming that the biopterin/neopterin balance regulates immune functions, which is strongly suggested by previous publications [4, 30, 31]. Either the supplementation with BH4 may beneficially restore normal anti-oxidative capacity of BH4 and iron homeostasis, or supplementation may further stimulate immune cell proliferation or activity and increase the autoimmune attack. To answer this question we used two models of autoimmune encephalomyelitis in mice (EAE) in two separate sequential experiments, the first with relapsing remitting EAE (RR-EAE; SJL mice) and the second with primary progressive EAE (PP-EAE; C57BL6 mice).

### Sapropterin (BH4) Treatment Aggravates EAE in Mice

**Fig. 2:**
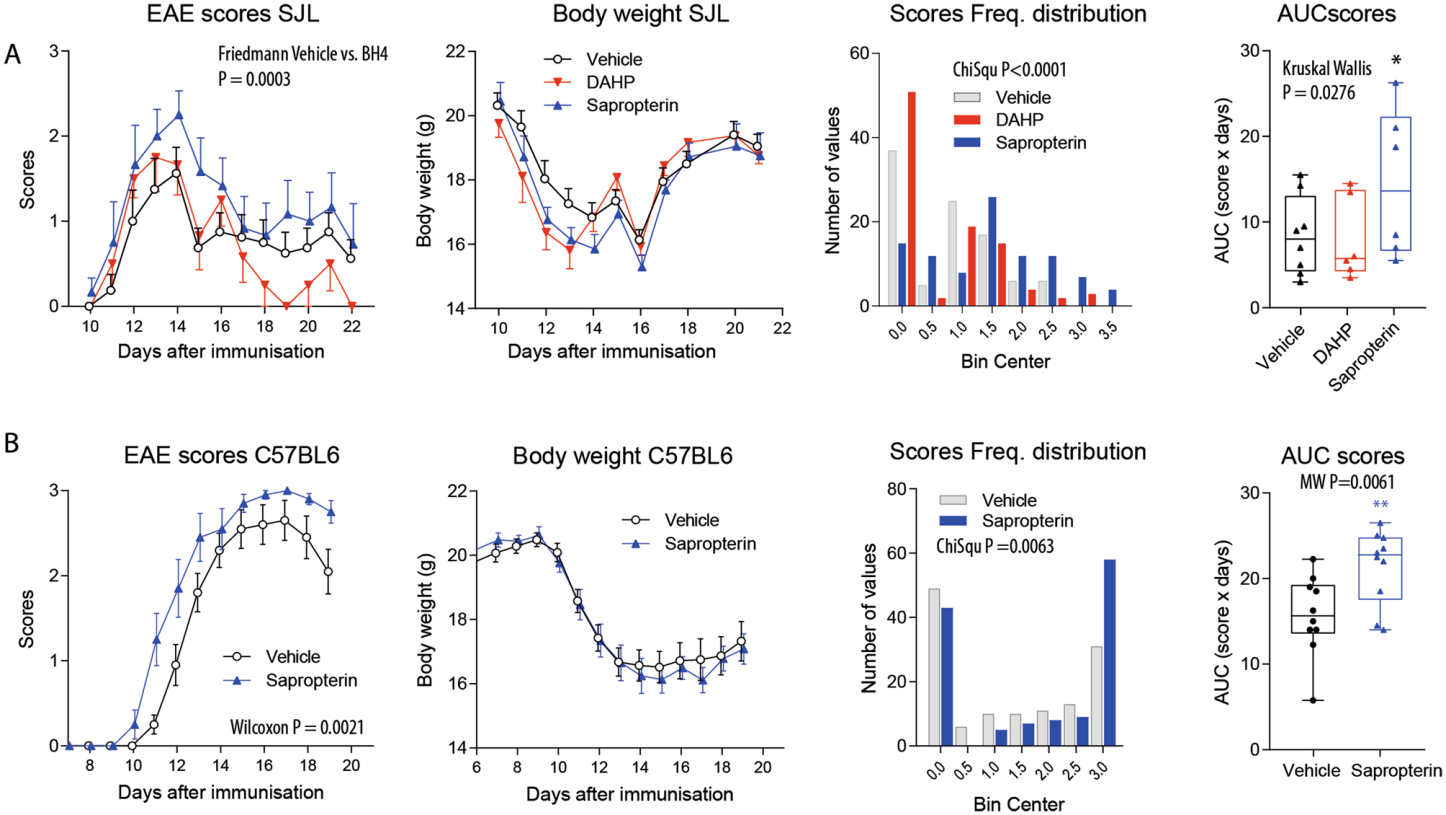
Effects of sapropterin medication on the disease severity in EAE mice.** a** Time courses of the clinical EAE scores (median ± siqr), body weights (mean ± SD), score frequency distribution, and AUCs of score of SJL/J mice in PLP-induced relapsing–remitting EAE. Mice were treated orally with vehicle (2% DMSO, *n* = 8), sapropterin (2 mg/day, *n* = 6), or DAHP (4 mg/day, *n* = 6) in the drinking water starting at the day of immunization. The score courses were analyzed using Friedmann statistics, the score frequency distribution using the chi-square test, and the AUCs of the scores were compared with the Kruskal Wallis test (*n* = 6–8 per group). **b** Time courses of the clinical EAE scores, body weights, score frequency distribution, and AUCs of EAE scores in C57Bl6/J mice in MOG-induced primary progressive EAE. Mice were treated orally with vehicle (*n* = 10) or sapropterin (*n* = 10) soaked cornflakes once daily (dosages as in **a**). The score courses were analyzed using Wilcoxon P statistics, the score frequency distribution using the chi-square test and the AUCs of the scores were compared with the Mann–Whitney *U* test (*n* = 10 per group). The box plots in **a** and **b** show the interquartile range, the line is the median, whiskers show minimum to maximum, each scatter is a mouse.

In the first experiment in SJL mice, mice were treated with sapropterin (BH4) or DAHP (GCH1-inhibitor) or vehicle perorally starting at the day of immunization (*n* = 6–8 per group). Sapropterin-treated mice reached higher maximum scores in SJL RR-EAE mice (Fig. 2a). DAHP oppositely reduced scores during the first relapse (Fig. 2a). Both drugs had no effects on body weights. The second experiment in C57BL6 PP-EAE mice (*n* = 10 per group) provided similar results (Fig. 2b). Indeed, sapropterin-treated mice had higher median scores, and the frequency of mice with high scores was increased. The C57BL6 experiment was done with vehicle and sapropterin only.

### BH4 Treatment Increases Infiltration of Immune Cells in the Spinal Cord in EAE Mice

**Fig. 3:**
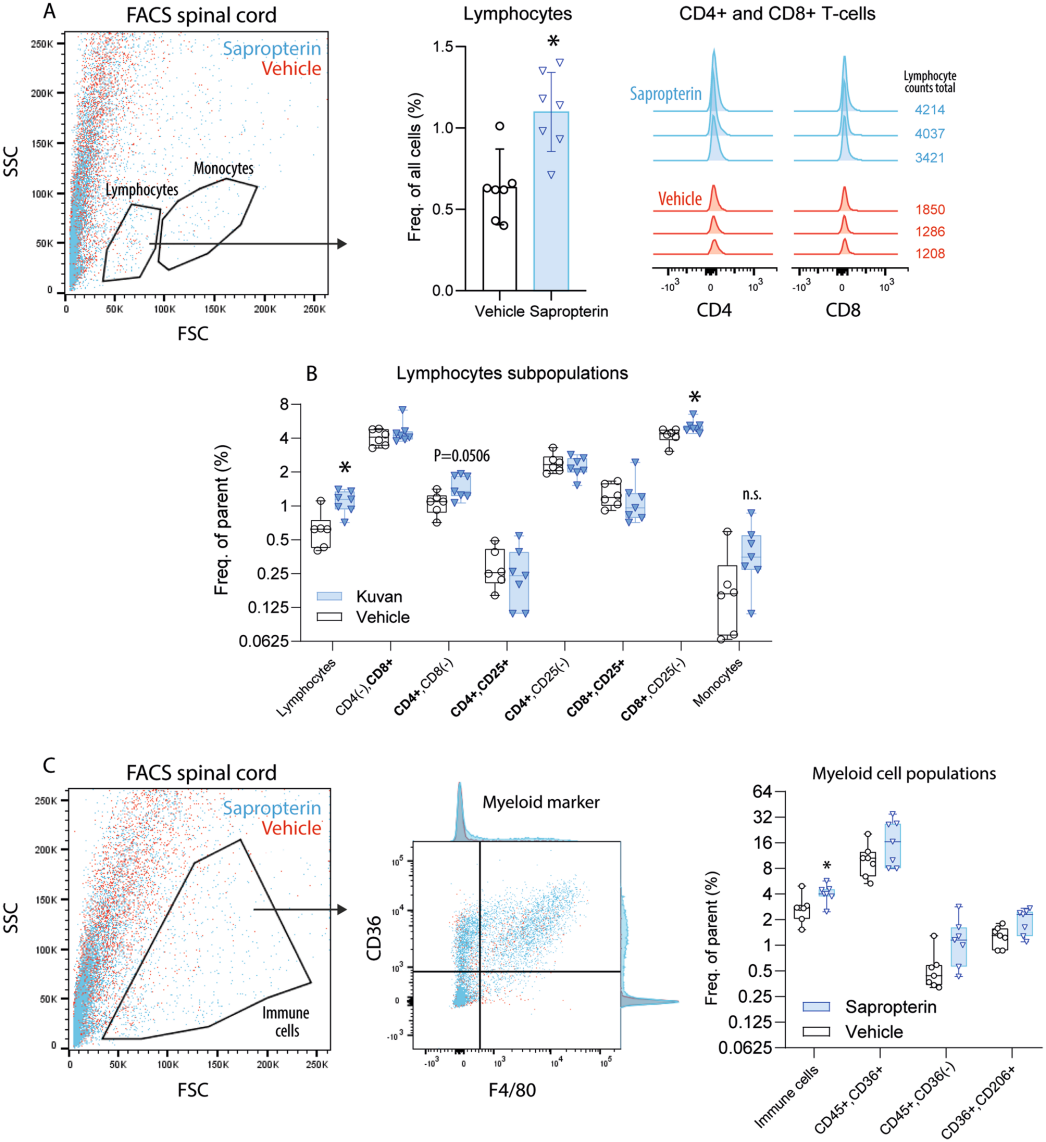
FACS analyses of immune cells in the spinal cord of EAE mice**. a** FACS analysis of lumbar spinal cord cells from vehicle or sapropterin (BH4) treated C57Bl6/J-EAE mice (*n* = 6–8 per group). The tissue was dissected 19 days after immunization and 300,000 cells were counted of each seven mice. The dot plot shows exemplary cell clouds gated according to forward scatter (FSC) versus sideward scatter (SSC). The gates show lymphocytes and monocytes according to size and granularity. The bar chart shows the frequency of lymphocytes as percentage of viable cells (compared per unpaired, 2-sided *t* test; *P < 0.05). CD4- and CD8-positive T-cell subpopulations are shown in the right panel histograms. **b** Quantification of lymphocyte subpopulations. Lymphocytes and monocytes were gated according to FSC and SSC, and lymphocytes subsequently according to CD4, CD8, and CD25. Data were compared with ANOVA for “population” × “treatment” and subsequent post hoc analysis for “treatment.” Please note the overall low numbers of T-cells as shown in the right panel in **a**. **c** FACS analysis of lumbar spinal cord cells as in **a**. Immune cells were further analyzed for F4/80 and CD36 (exemplary dot plot, middle). The box plot shows the frequency of myeloid subpopulations (as percentage of all immune cells). The box is the interquartile range, whiskers show minimum to maximum, the line is the median. Each scatter is one mouse.

**Fig. 4:**
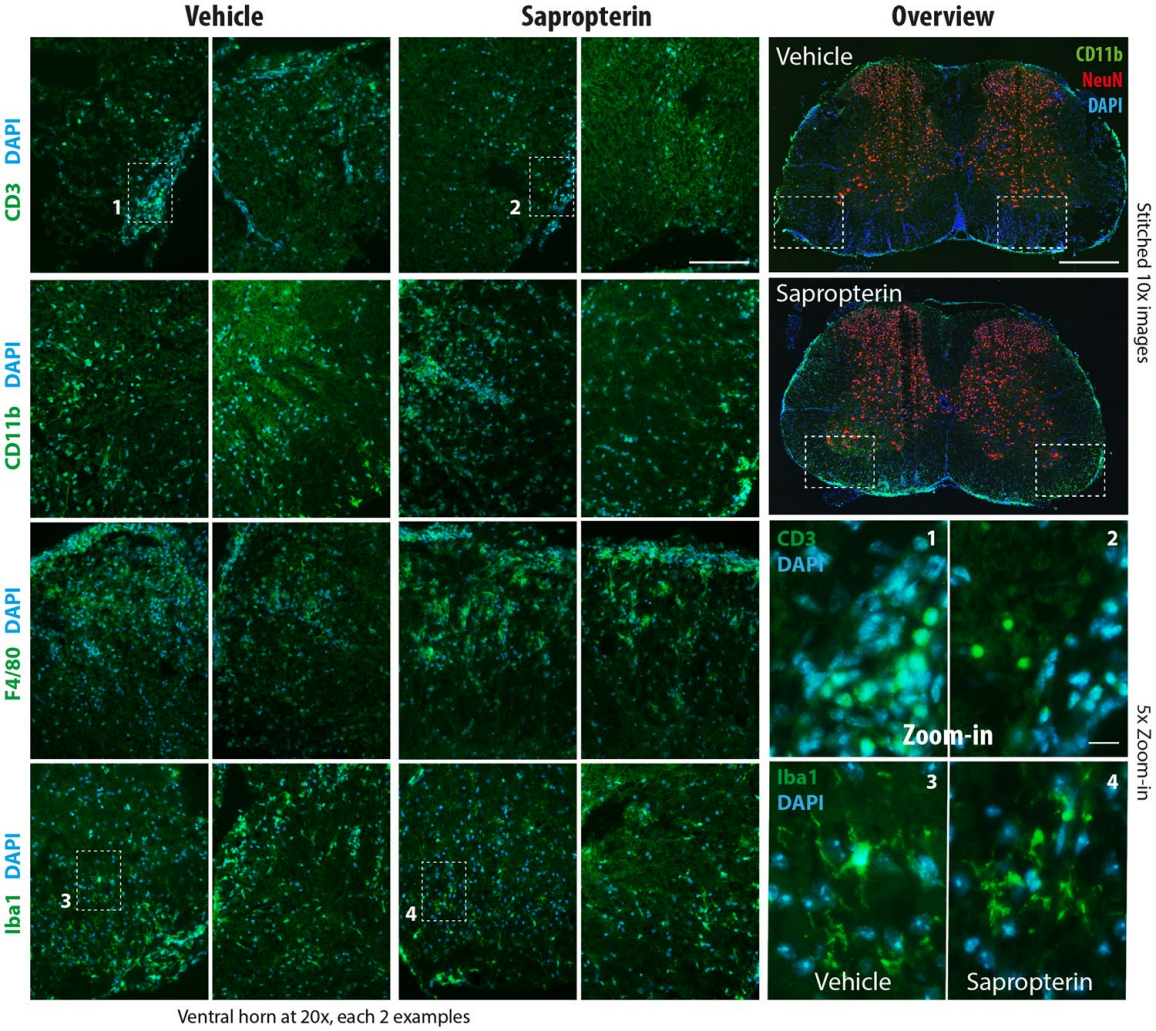
Immunofluorescence analyses of immune cell infiltration of the spinal cord in EAE mice. C57Bl6/J mice were immunized with MOG and treated with vehicle or sapropterin (BH4) starting at the day of immunization. The tissue was prepared 19 days after immunization. Myeloid cells were stained with anti-CD11b, Iba1, and F4/80. T-cells were identified via anti-CD3. NeuN was used as neuronal counterstain, and DAPI to label nuclei. For the overviews, tiled images were captured and stitched. Scale bars are 500 µm and 20 µm (zoom in). Exemplary images of *n* = 3 mice per group. IF panels and quantification in Suppl. Figs. 2-5.

The observed increase of EAE scores in sapropterin-treated mice was associated with higher numbers of T-cells infiltrating the lumbar spinal cord white matter, which was revealed by FACS analyses (Fig. 3a, b, C57BL6). CD4 + and CD8 + (CD25(−) T-cell subpopulations were significantly increased (Fig. 3b). Immunofluorescent analyses of CD3+ T-cells (Fig. 4 top panel, Suppl. Fig. 2, C57BL6) suggested deeper infiltrates but the quantification did not reach statistical significance (Suppl. Fig. 2).

FACS results for individual myeloid cell populations were not significant (Fig. 3c), but immunofluorescence studies suggested larger hotspots of cellular invasion with staining of F4/80 (Fig. 4, Suppl. Fig. 3 n.s.). CD11b and Iba1 immunofluorescence quantifications (Suppl. Figs. 4 and 5) were not significantly different.

**Fig. 5:**
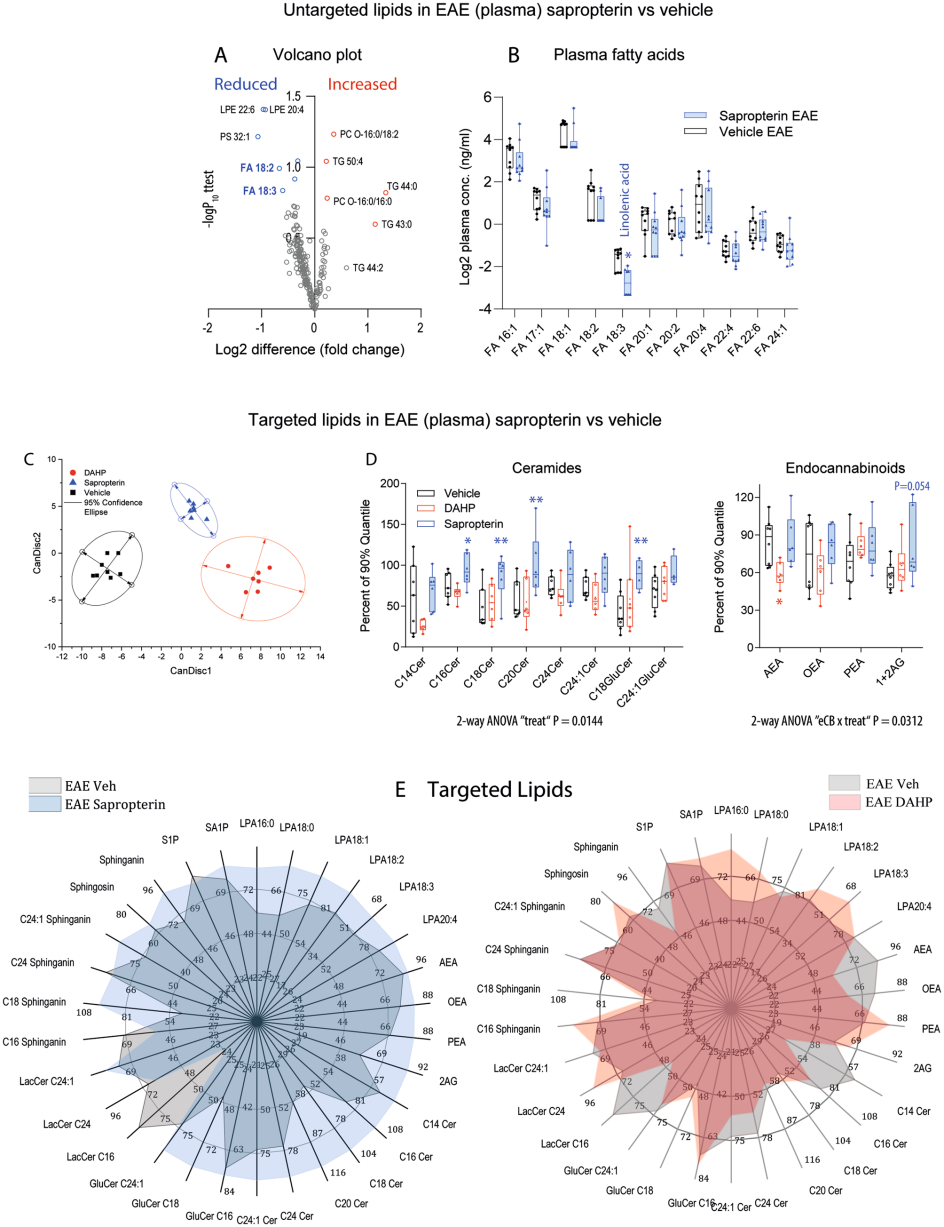
Targeted and untargeted lipidomic analyses of bioactive and metabolic lipids in plasma in EAE mice. **a** Volcano plot show the log2 difference (= fold difference; *X*-axis) of metabolic plasma lipids between sapropterin and vehicle-treated EAE mice versus the –log10 of the *P* value of the *t* test (*Y*-axis). Lipids that were reduced in sapropterin-treated mice appear on the left side of the *Y*-axis, increased lipids on the right side (*n* = 10 per group). **b** The box/scatter plot shows the log2 transformed levels of fatty acids. The data represent the ratios of the mass spectrometry peak areas normalized by the peak areas of the respective standard. The box represents the IQR, whiskers show minimum to maximum, the line is the median. Each scatter is a mouse (*n* = 10 per group). Data were compared with two-way ANOVA for “fatty acid × treatment” and subsequent post hoc analysis for treatment using an adjustment of alpha according to Šidák. **c** Canonical discriminant score plots of the first discriminant factors CanDis1 and CanDisc2 for plasma lipids using 28 lipid species of five classes as input. The plasma concentrations were obtained from SJL/J mice immunized with PLP and treated orally with vehicle (2% DMSO), sapropterin (2 mg/day) or DHAP (4 mg/day) via the drinking water starting at the day of immunization (*n* = 6–8 per group). The final blood sample for lipid analyses was obtained 22 days after immunization. Lipids encompassed ceramides, hexosylceramides, sphingolipids, endocannabinoids, and lysophosphatidic acids (individually presented in Suppl. Fig. 4C). The dots show individual mice. The ellipses show the 95% confidence. **d** Box/scatter plots of normalized ceramides and endocannabinoids in vehicle, sapropterin (BH4), or DAHP-treated SJL/J-EAE mice as in **a**. Lipids were normalized as percentages of the 90% quantile (raw concentrations shown in Suppl. Fig. 4c). The box represents the interquartile range, whiskers show minimum to maximum, the line is the median. Each scatter is a mouse. Data were compared with two-way ANOVA for “lipid × treatment” and subsequent post hoc analysis for treatment using an adjustment of alpha according to Šidák (*n* = 6–8 per group, *P < 0.05, **P < 0.001). **e** Polar plots show the mean normalized levels (percentages of the 90% quantile) of multiple lipid species analyzed via targeted LC–MS/MS analyses in plasma of SJL/J-EAE mice treated as in **a**. Most ceramides and some lysophosphatidic acids (LPAs) were increased in sapropterin-treated mice.

Proliferation of peripheral T-cells, T-cell subpopulations and myeloid cells in the spleen were not affected by sapropterin or DAHP treatment as compared to vehicle treated EAE mice (Suppl. Fig. 6, SJL and C57BL6), suggesting that oral BH4 treatment did not reinforce peripheral T-cell proliferation but might be permissive for CNS infiltration.

**Fig. 6:**
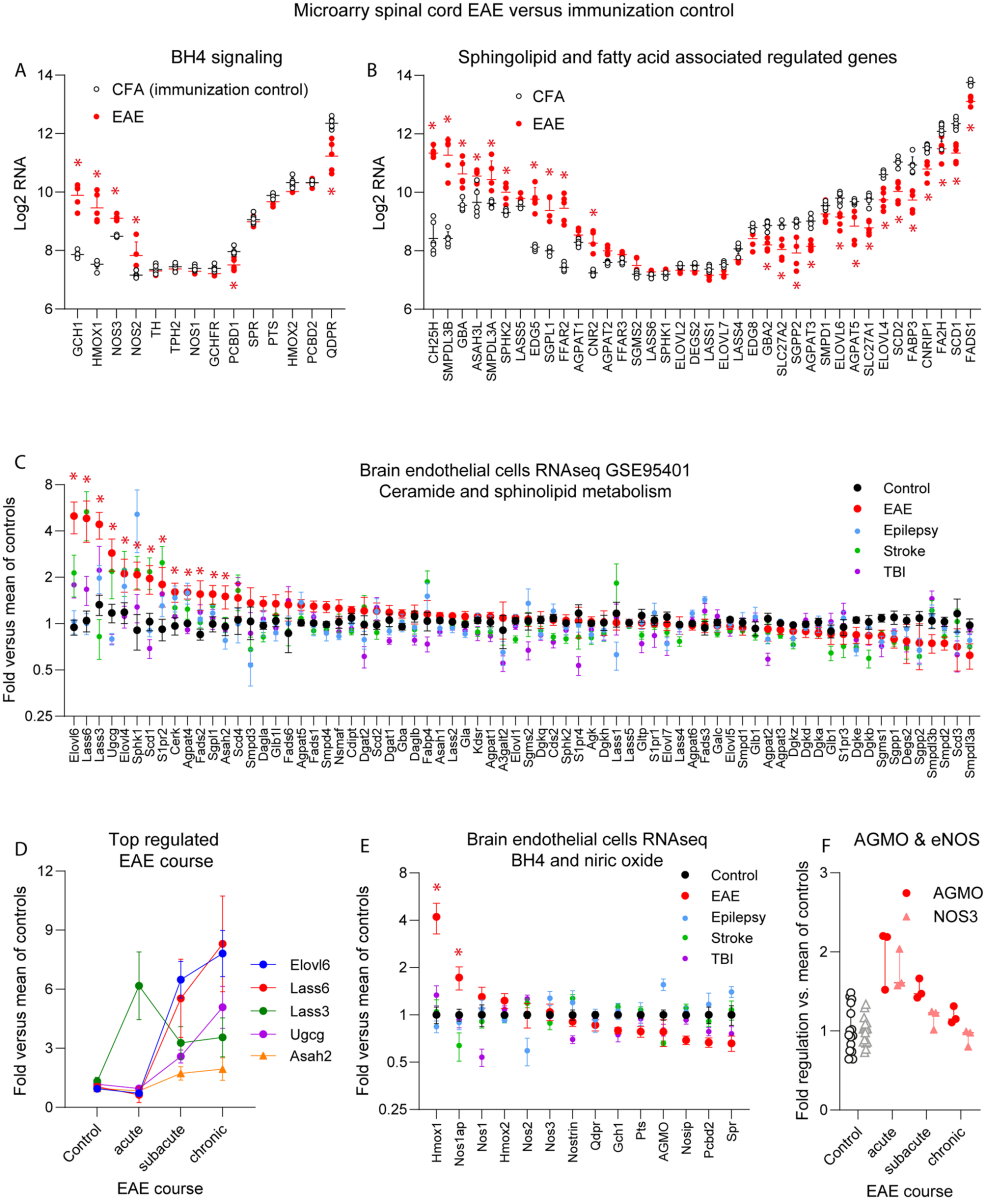
RNA analyses of EAE-regulated genes in spinal cord and in brain endothelial cells in disease models. **a** Scatter plots of BH4 or nitric oxide associated genes (microarray) in the spinal cord of C57BL6 EAE mice versus control mice (CFA without MOG) 16 days after immunization at the flare of the disease (GEO dataset GSE60847). *Significant at FDR < 0.05. **b** Scatter plots of sphingolipid and fatty acid associated regulated genes (microarray) in the spinal cord of C57BL6 EAE mice versus control mice (CFA without MOG, *n* = 6 per group) 16 days after immunization (GSE60847). Genes were text filtered for, e.g., sphingolipid, lipid, fat, or fatty, to find lipid-pathway genes and sorted according to fold difference and *P* value < 0.1. *Significant at FDR < 0.05. **c** GSE95401 RNAseq dataset [41] analysis of genes involved in metabolisms of ceramides and sphingolipids in brain endothelial cells in control mice, epilepsy, EAE, stroke, and traumatic brain injury (TBI). All controls were pooled (*n* = 16) and acute, subacute, and chronic phases (each *n* = 3) were pooled per disease. *Significant at FDR < 0.05; EAE versus control. **d** Disease-course dependent top upregulated genes in the EAE model (data as in **c**). ELOV, elongase; LASS, ceramide synthase; UGCG, UDP-glucose ceramide glucosyltransferase; ASAH, neutral ceramidase). **e** Data as in **c** show genes involved in tetrahydrobiopterin (BH4) and nitric oxide biosynthesis and BH4-dependent enzymes that were expressed in brain endothelial cells in controls and disease models. *Significant at FDR < 0.05; EAE versus control. **f** Disease-course-dependent regulation of BH4-dependent AGMO and endothelial NOS (eNOS/NOS3) in brain endothelial cells in EAE (data as in **c**).

### Sapropterin and Lipid Signaling Hypothesis

Based on our results, we had two major hypotheses how BH4 might affect CNS invasion with immune cells (i) high levels of circulating BH4 may activate endothelial NOS (NOS3) at the BBB, facilitating BBB breakdown [16] or (ii) BH4 may lead to changes of lipid homeostasis thereby affecting barrier functions. The second hypothesis was based on its coenzyme function for AGMO [24] and our previous studies in colitis mice where BH4/DAHP altered lysophosphatidic acids, ceramides and the endocannabinoid, arachidonoylglycerol [7], all clinically relevant for MS [32–36]. Because NO likely has dual effects in EAE [16, 37] we opted for the putative lipid-paths (Fig. 5; Suppl. Fig. 7) and regulations of lipid-associated genes in bulk EAE tissue and in brain endothelial cells (Fig. 6). It is of note that eNOS is highly expressed in endothelial cells [38], and AGMO was expressed and upregulated in EAE brain endothelial cells (Fig. 6) and it was also strongly expressed in choroidal cells at the brain-to-CSF barrier (Suppl. Fig. 7).

### Sapropterin (BH4) Treatment Raises Long-Chain Ceramides and 2-AG in EAE Mice

A lipidomic screen of plasma and lymph nodes of EAE mice treated with sapropterin or vehicle revealed lipid alterations of metabolic lipids in plasma (Fig. 5) but not in the lymph nodes in sapropterin versus vehicle treated EAE mice (Suppl. Fig. 8A, B). In plasma, polyunsaturated fatty acids, in particular linolenic acid (FA18:3), were reduced in sapropterin treated mice (Fig. 5b, c). It is of note, that feeding of mice with this omega-3 lipid was shown to preserve the BBB in EAE mice by restoring the gating properties of TASK1 two-pore potassium channels [42].

Targeted LC-MS/MS lipidomic analyses were used to assess bioactive signaling lipids (Fig. 5d–f; Suppl. Fig. 8C). Using lipid species of five classes (28 different species) as input, canonical discriminant analysis clearly separated groups and allowed a > 95% correct prediction of group membership based on the first two CanDisc scores (Fig. 5d). The clear separation was based mainly on ceramides of different chain lengths. Ceramides were increased in sapropterin-treated EAE mice as compared to vehicle-treated mice, with opposite regulations with DAHP (Fig. 5e), suggesting that sapropterin aggravated ceramide upregulations, that occur at the BBB in EAE. Re-analyses of RNA data (Fig. 6) suggested that the ceramide increased was caused by upregulations of genes involved in ceramide de novo synthesis, namely LASS6/CERS6 and LASS3/CERS3 (Fig. 6).

Sapropterin treatment was additionally associated with inverse regulations of endocannabinoids. Sapropterin increased 2-AG, whereas DAHP reduced anandamide, AEA (Fig. 5e, right). The endocannabinoid changes may reflect drug-dependent differences of disease activity that was higher with sapropterin, or/and may point to a BH4-mediated regulation of endocannabinoid metabolism. We have shown previously that the 2-AG precursor, 2-AG-ether (noladin ether), is a substrate of AGMO *in vitro* [7], which may link BH4 to 2-AG metabolism. The increase would suggest a higher rate of production via ether intermediates in EAE mice, which cannot be tested experimentally *in vivo* because of the instability of ether-eCB intermediates. Functionally, 2-AG has both pro- and anti-inflammatory effects depending on the disease context, environment, and abundance of cannabinoid receptors CB1 and CB2 [43–45].

Polar plots in Fig. 5f give an overview of multiple bioactive lipid species and reveal an increase of ceramides and unsaturated LPAs in sapropterin-treated mice, whereas lipids were mostly normal in DAHP-treated mice. Scatter plots of the concentrations of individual mice are shown in Suppl. Fig. 8C. Ceramide homeostasis is crucial for the maintenance of plasma membrane integrity [46] and increased levels suggest leaky membranes and dysfunctions of lysosomal breakdown [47]. High ceramides in plasma likely reach the endothelial cells and might affect brain barrier functions.

### Increased Expression of Genes Involved in Ceramide Biosynthesis in EAE

High plasma ceramides may arise from upregulations of genes involved in ceramide production or breakdown. To address transcriptional changes, we reanalyzed our previous microarray data of bulk spinal cord EAE versus naïve mice [39] (GEO dataset GSE60847) and RNAseq data of brain endothelial cells in injury models including EAE (GSE95401 [41]). Genes were searched according to descriptions to find genes involved in BH4 synthesis and signaling, or in genes involved in sphingolipid and fatty acid metabolism and their receptors.

The RNA studies of the lumbar spinal cord showed that GCH1 was increased as expected as well as NOS2 and NOS3 (Fig. 6a). In addition, the data revealed some previously not recognized EAE-dependent deficits of fatty acid desaturases (FADS), stearoyl CoA desaturases (SCD1, SCD2) and fatty acid elongases (ELOVL) (Fig. 6b), but increased glucocerebrosidase alpha (GBA) that degrades glucosylceramides (Fig. 5a). The loss of desaturases, in particular FADS1, which is involved in FA18:2 and FA18:3 generation (linoleic and linolenic acid), is remarkable in light of the protective preserving effects of these omega-lipids in EAE and/or human MS [23, 40].

Lipid deregulations contributing to BBB breakdown were further strongly supported by RNA sequencing data of the Geo dataset, GSE95401 [41], in which gene regulations were analyzed in brain endothelial cells in disease models with a profound disruption of the blood brain barrier, namely EAE, epilepsy, stroke, and traumatic brain injury. We focused on genes involved in bioactive lipid metabolism or BH4 production and coenzyme functions (Fig. 6c–f). The data show a remarkable increase of genes involved in ceramide synthesis, most strongly ceramide synthase 6 (LASS6/CERS6) and LASS3. The others were elongases, ELOVL6 and ELOVL4, and the ceramide glucosyltransferase, UGCG (Fig. 6d, e). AGMO was increased in dependence of the disease stage (Fig. 6g) and localized at the brain-to-CSF barrier (Suppl. Fig. 7). The observed gene upregulation in brain endothelial cells including LASS6 and LASS3 (GSE95401; [41]) suggested that the observed increases of ceramides may arise from lipid-gene deregulations at the BBB. The data strengthen the idea that unfavorable effects of sapropterin were caused at least in part via deregulation of ceramides.

## Discussion

We show in the present study that patients with MS at different stages have reduced serum biopterin levels and temporarily increased neopterin concentrations suggesting high turnover and consumption, which was supported by a drop of biopterin *ex vivo* upon immune stimulation of human whole blood. Previous studies show that BH4 assists in NRF2 (nuclear factor erythroid 2-related factor 2) activation [3, 13], which is a key mechanism of recently favored MS drugs, fumaric acid esters [17]. Hence, sapropterin supplementation might foster NRF2 with beneficial clinical outcome. On the other hand, a recent paper suggested that BH4 boosts autoimmune responses [4]. In agreement with the latter, we found that oral sapropterin (BH4, Kuvan®) treatment in mice mildly aggravated immunization-evoked EAE and increased the numbers of infiltrating T-cells in the spinal cord, however without effect on peripheral immunocyte numbers and subtypes in blood and spleen, but with profound increases of systemic ceramides that are known to contribute the MS/EAE pathophysiology [49, 50] and to the invasion of immune cells [48]. The results suggest that sapropterin facilitated the invasion of immune cells into the CNS via changes of lipid homeostasis possibly affecting barrier functions and/or attachment properties of immune cells [48].

Sapropterin is a safe and well-tolerated drug for replenishment of BH4 deficiency in phenylketonuria patients [10, 51], and it did not increase T-cell proliferation in our study. However, our data point to a putative risk and need for caution in patients with autoimmunity. Although mechanistically different, the conclusion is in agreement with the previous study of Cronin et al. showing that BH4-deficient T-cells have a proliferation defect [4], which was attributed to a defect of mitochondrial iron transport [4].

Mechanistically, we focused on alterations of lipid signaling molecules and metabolic lipids rather than iron-mediated direct effects on T-cells because we have previously observed lipid alterations in a colitis model in dependence of BH4 [7], AGMO is abundant in epithelial barriers and known as modulator of lipid homeostasis in immune cells [24]. In addition, re-analysis of RNAseq data of brain endothelial cells [41, 52] revealed robust deregulations of genes involved in ceramide synthesis at the BBB in the EAE model, particularly sixfold increases of ceramide synthase 6 and 3 (Fig. 6).

The analyses of immune cells and lipids led us to the hypothesis that sapropterin had a permissive effect for invasion of immune cells into the CNS, and previous studies suggest that the effect may arise from alterations of ceramides in membrane microdomains that affect integrin clustering [48, 53, 54]. However, our studies are limited in that we did not directly measure BBB leakage in dependence of sapropterin treatment. Hence, our studies are descriptive and do not proof that sapropterin alters BBB integrity or attachment of immunocytes. Further studies are needed to address our hypothesis experimentally.

There is some evidence from previous studies in mice and humans that ceramides are increased in EAE [33, 55] and MS [50, 56, 57] and contribute to the disruption of the BBB [21, 58]. In a study using knockout mice or inhibition of acidic sphingomyelinase (ASM), it was suggested that high levels of ceramides are caused by over-activation of ASM [59, 60], which produces ceramides in the lysosome via sphingomyelin degradation. Other studies reported upregulations of specific ceramide synthases [21, 33] that produce ceramides de novo from sphinganines [61], in line with the RNAseq re-analysis of the Geo dataset GSE95401. The observed disease protection of ASM knockout mice against immunization-evoked EAE [59] may result from an overall lowering of ceramides. High levels of ceramides may reflect disease activity [33, 55], not necessarily directly mediated through BH4. ASM activity in humans was not associated with the activity of MS lesions in patients although overall ASM activity in blood was higher in MS patients than healthy controls [62]. Hence, high ceramides in blood/plasma in MS/EAE likely originate mostly from de novo synthesis via ceramide synthases and are contributed by the ASM path. Reanalysis of RNAseq data (GSE95401) of brain endothelial cells identified upregulations of ceramides synthase 6 and 3 (LASS6, LASS3) in models of BBB disruption particularly EAE [41], which is remarkable in light of the well described causative role of LASS6 in EAE pathology [33, 63]. It has to be considered that loss of ceramide homeostasis affect further lipid species and downstream glucosyl- and lactosylceramides that are a major topic of recent neurodegenerative research [64–66].

In contrast to ceramides, epidemiology studies suggest beneficial effects of PUFAs in MS [68], in particular linolenic acid, which was neuroprotective in EAE mice [23], and is generated via cytochrome B5 dependent stearoyl CoA desaturases and other fatty acid desaturases. Indeed, gene expression analysis of spinal cord from EAE versus naïve mice revealed reduced levels of fatty acid desaturases and stearoyl-CoA desaturases (FADS1, SCD1, and SCD2) in EAE mice. Desaturases (DEGS and SCD subtypes) were also low in brain endothelial cells in models of robust BBB disruption. SCD-mediated desaturation is carried out with help of cytochrome B5 and is dependent on iron cycling. Like AGMO, it is a transmembrane ER enzyme that generates linolenic acid (FA18:3) from linoleic acid (FA18:2) (among others), hence linking iron, BH4, and lipid metabolism. Incorporation of PUFAs into biological barriers increases membrane fluidity and facilitates the insertion of receptors [69], whereas high ceramides disrupt barrier functions [70]. Omega-3 lipids have been proposed to be putative supportive nutrients in MS [40, 71] and other inflammatory diseases. Hence, we believe that the deficiency of linolenic acid that manifested under sapropterin diet contributes to the sapropterin-associated aggravation of the disease. Linolenic acid plays an important role in BBB maintenance in the EAE model [42].

In addition to deregulations of ceramides and linolenic acid, sapropterin therapy in EAE was associated with an increase of plasma levels of the endocannabinoid, 2-AG. BH4/AGMO may be linked to ceramide synthesis in the ER via generation of precursor lipids [24]. This also hold true for 2-AG, which can be generated via AGMO mediated cleavage of 2-AG-ether (noladin ether). The clinical relevance of this path of 2-AG generation in the context of EAE is unknown. High 2-AG levels in sapropterin treated EAE may be a compensatory mechanism via CB2 to counteract the immune activation. In support, inhibition of 2-AG breakdown via inhibitors of monoacylglyerol lipase attenuated inflammation in a brain trauma model [45] or in models of inflammatory pain [43, 72]. On the other hand, 2-AG has unfavorable effects for example in the context of inflammation in obesity [73, 74] and liver disease [75, 76]. It is of note, that ceramides and endocannabinoid paths are inter-connected [67]. Hence, the lipid pattern under sapropterin rather than an individual lipid is likely to determine the outcome.

Overall, the lipid alterations and clear discrimination of treatment groups based on lipids appear to be too strong to be mechanistically not associated with sapropterin. Lipid alterations may arise from changes of BH4-cofactor availability for the ER-localized lipid-metabolizing enzyme, AGMO. We observed high expression of AGMO in a LacZ reporter mouse at the brain-to-CSF barrier in the ependymal epithelium (Suppl. Fig. 7), whereas endothelial NOS is highly expressed in brain endothelial cells and it is important for BBB functions [16]. AGMO was also expressed in brain endothelial cells and upregulated in EAE (Fig. 6). Hence, BH4 may act at two crucial barrier sites in the CNS and result in a permissive effect on immune cell invasion of the CNS, by acting as a coenzyme or via alterations of lipid homeostasis.

It is of note that oral sapropterin treatment in mice did not have such permissive effects on T-cell infiltration of the lamina propria in dextran sulfate sodium (DSS) evoked colitis model [7]. The intestinal epithelial barrier and BBB differ in microenvironments, mesodermal versus endodermal origin and the molecular composition of the tight junctions [77] and likely lipid composition of the membranes. The lipid compositions have not yet been directly compared, but gene expression data show distinct gene enrichments in peripheral versus brain endothelial cells [41, 52]. Omega-3 lipids are protective at both sites [40, 78], 2-AG is pro or anti-inflammatory in the gut and slows down intestinal transit [79–82], and high ceramides are detrimental at both sites [33, 70]. We hypothesize that the differences in the outcomes rely in the models per se. EAE is autoimmune driven whereas DSS disrupts the mucous layer and gives microbiota access to the intestinal wall. Oral sapropterin might also directly affect the gut microbiome.

It is important that sapropterin treatment did not increase T-cell proliferation in the periphery. Hence, it was not a general “immune boost” as suggested by the study of Cronin et al. [4] but still, it aggravated autoimmune CNS disease in the EAE model. We infer that oral sapropterin is safe as supplementation, albeit possibly with caution in autoimmune-directed CNS disease.

## Methods

### Patients with Multiple Sclerosis and Healthy Controls

Human samples and biographic data were available from an observational cross-sectional investigation including 102 multiple sclerosis (MS) patients (31 men, 71 women) as described in [32, 83] (Suppl. Table 1A). They were consecutively recruited from outpatients and inpatients of the Department of Neurology of the Goethe University Hospital Frankfurt, Germany. Data and blood collection was part of the local bio-banking project (Neurological Department of the Goethe University, Frankfurt). The diagnosis of MS was based on ICD10 criteria. Fourteen additional patients with serious disease courses were recruited and observed up to 3.5 years for time course analyses. The patients participated in clinical efficacy studies of fingolimod or natalizumab (NTZ) (Suppl. Table 1B).

To cover the whole period, control samples were analyzed from four consecutive cohorts of healthy subjects (HC). The first encompassed 117 men and 233 women with a mean age of 28 ± 8 years (range 18–57 years, plasma samples). The second were 118 men and 183 women, aged 25 ± 6 years (range 18–57 years, serum samples), the third were 108 m, 217 f with a mean age of 35 ± 12.8 years (range 18–68 years, plasma samples), and the last cohort comprised each 25 men and women above 50 years of age (mean ± SD: 62.9 ± 8.4 years, range 50–79, plasma samples). HC cohorts 1 to 3 were recruited through the Occupational Health Service at the University Hospital of Frankfurt, Germany. HC cohort-4 was recruited from family, friends, and colleagues.

For the whole blood assay, venous blood of healthy donors was sampled in K^+^-EDTA tubes as described in [29], each split into two samples, one stimulated with 10 µg/ml LPS, the other unstimulated and kept in a 37 °C water bath for the indicated times, and biopterin was analyzed in plasma.

The studies were approved by the Ethics Committee of the Medical Faculty of the Goethe University and adhered to the Declaration of Helsinki. Informed written consent was obtained from each participating subject. Venous blood samples were collected to K^+^ EDTA tubes or in serum tubes and centrifuged at 3000 rpm for 10 min. Plasma and serum were frozen at − 80 °C up to analysis.

### Animals and Drug Treatments

Female 10–12-week-old SJL/J mice (Charles River, Germany) were used for study of relapsing–remitting EAE, and C57Bl6/J mice (Charles River, Germany) were used for the study of primary progressive EAE. Mice were housed at 2–4 mice per cage at constant room temperature (21 ± 1 °C) under a regular light/dark schedule with light from 7:00 A.M. to 7:00 P.M. Food and water were available ad libitum.

For the treatment of SJL/J-EAE mice, DAHP (Sigma #D19206; 4 mg/day, ~ 200 mg/kg/day; *n* = 6) or BH4 (Sigma #T4425; 2 mg/day, ~ 100 mg/kg/day; *n* = 6) were dissolved in the drinking water with 2% DMSO. Control animals received the respective vehicle (*n* = 8).

In C57Bl6/J-EAE mice, BH4 was administered perorally once daily in cornflakes soaked with 10% sucrose/5% ethanol in water (*n* = 10 BH4, *n* = 10 vehicle). Treatments started at the day of immunization. Control animals received the respective vehicle.

AGMO LacZ reporter mice were used to assess the localization of AGMO in the brain and were created according to EUCOMM gene targeting strategy [84].

The experiments were approved by the local Ethics Committee for Animal Research (Darmstadt, Germany) and adhered to the European guidelines and to those of GV-SOLAS for animal welfare in science and agreed with the ARRIVE guidelines.

### EAE Model

SJL/J mice were immunized using the Hooke Kit™ 2110PLP139-151/CFA emulsion PTX (EK-2120, Hooke Labs, St Lawrence, MA), which contains 200 µg of peptide 139–151 of myelin proteolipid protein (PLP) emulsified in 200 µl complete Freund’s adjuvant (CFA). The emulsion was injected subcutaneously (s.c.) at two sites followed by two intraperitoneal (i.p.) injections of 200 ng pertussis toxin (PTX) in phosphate-buffered saline (PBS), the first 1–2 h after, and the second 24 h after PLP_139-151_.

C57Bl6/J mice were immunized using Hooke Kit™ MOG_35-55_/CFA emulsion PTX (EK-2110), which contains 200 µg of a peptide 35–55 (amino acids) of myelin oligodendrocyte glycoprotein (MOG) in 200 µl CFA (Hooke Labs, USA). Injections of the emulsion and PTX were done as described above.

EAE scores and body weights were assessed daily by an observer blinded for drug treatments to evaluate the disease severity and extent of motor function deficits: score 0, normal motor functions; score 0.5, distal paralysis of the tail; score 1, complete tail paralysis; score 1.5, mild paresis of one or both hind legs; score 2, severe paresis of one or two hind legs; score 2.5, complete paralysis of one hind leg; score 3, complete paralysis of both hind legs; score 3.5, complete paralysis of hind legs and paresis of one front leg.

Blood and tissue samples were obtained at the end of the clinical observation time, 19–22 days after immunization. Blood was collected in K^+^ EDTA microtubes (EDTA K^+^ Microvette Sarstedt), centrifuged at 3000 rpm for 10 min and stored in standard Eppendorf caps at − 80 °C until analysis. Tissue samples were snap frozen on dry ice and stored at − 80 °C until lipid analysis or they were freshly prepared for FACS.

### FACS Analysis of Surface Marker Proteins

Single-cell suspensions were prepared from the spleen, and the lumbar spinal cord. Tissues were rapidly dissected, treated with lysis buffer (DMEM/accutase (PAA) 1:1, collagenase (3 mg/ml, Sigma), DNAse I (1U/ml, Promega)) for 30 min at 37℃, followed by mechanical disruption, which was done by forcing the tissue through a nylon mesh with 70 μm pore size (Cell Strainer, BD). Cell suspensions were mixed with 1 ml erythrocyte lysis buffer for 10 min at room temperature and CD16/32 blocking antibody (Fc$\gamma$ RII/III receptor blocker, BD) for 15 min on ice. For staining of cell surface antigens, cells were incubated for 20 min at room temperature in staining buffer with the respective fluorochrome-labeled antibodies (Suppl. Table 2) and were then counted with a flow cytometer (BD FACS Canto II). FACS scans were analyzed with FlowJo 10.6. For all FACS assays, antibody concentrations followed the recommendations of the manufacturers and the controls were FITC, PE, or APC-conjugated rat IgG.

### Immunofluorescence Analyses and LacZ Histology

A subset of mice was used for histology. Mice were terminally anaesthetized with isoflurane and cardially perfused with cold 0.9% saline, followed by 4% paraformaldehyde (PFA) in 1× PBS for fixation. The lumbar spinal cord was excised, post-fixed in 4% PFA for 2 h, cryoprotected overnight in 20% sucrose at 4 °C, embedded in tissue molds in cryomedium and cut on a cryotome (12 µm). Slides were air-dried and stored at − 80 °C. After thawing, slides were immersed and permeabilized in 1× PBS with 0.1% Triton-X-100 (PBST), then blocked with 3% BSA in PBST, subsequently incubated overnight with the first primary antibody in 1% BSA (Suppl. Table 2) in PBST at 4 °C, washed and incubated with the secondary fluorochrome-labelled antibody (Alexa488 or Cy3) for 2 h at room temperature. The procedure was repeated for further antibody pairs, followed by 10-min incubation with 1 µg/ml DAPI and embedding in Fluoromount (eBioscience).

For beta galactosidase (LacZ) visualization in tissue sections of AGMO-LacZ reporter mice, cryosections were postfixated for 5 min in 2% PFA, washed in 1× PBS with 2 mM MgCl_2_ and 3 times in washing buffer containing detergent (1× PBS/2 mM MgCl_2_ with 0.1% sodium deoxycholate, 0.02% Nonidet P40, pH 7.5) for 5 min at room temperature. Slides were then incubated at 37 °C with the staining solution consisting in 0.5 mg/ml nitrotetrazolium blue chloride (NTB), 5 µg/ml phenazine methosulfate (PMS) in detergent washing solution. The incubation time was adjusted to the tissue. The reaction was stopped by washing the slides 3× in 1× PBS for 5–10 min. Slides were counter-stained with eosin, dehydrated in increasing ethanol concentrations and xylene before embedding in Pertex mounting medium.

Tiled images were captured (× 10 objective lens) on an inverted fluorescence microscope (BZ-9000, KEYENCE, Germany) and were stitched using the Keyene’s software to cover the complete spinal cord. Filter and acquisition parameters were set to assure comparability. Subsequently, higher magnification images (× 20 objective lens) of the gray-to-white matter border were obtained of various regions. Immunoreactive cells were quantified using the particle counter plugin of FIJI ImageJ after background subtraction and threshold setting according to automatic algorithm implemented in FIJI. Zoom-in images (× 5) were created from regions of interest. The area covered by immunoreactive cells relative to the total area (which was identical in all images) was used to assess treatment effects. Three or more sections were analyzed per mouse.

### Culture of Splenocytes

Spleen tissue was rapidly dissected, treated with lysis buffer (DMEM/accutase (PAA) 1:1, collagenase (3 mg/ml, Sigma), DNAse I (1U/ml, Promega)) for 30 min at 37 °C, followed by mechanical disruption, which was done by forcing the tissue through a nylon mesh with 70 μm pore size (Cell Strainer, BD). Cell suspensions were washed, resuspended in PBS, and the cell number was counted with a Neubauer chamber. Cells (5 × 10^5^) were plated, cultured in RPMI1640-GlutaMax medium (Gibco™, Life technologies) and restimulated with 25 ng/ml IFN$\gamma$ for 24 h.

### Griess Assay of Nitric Oxide

The concentration of nitrite/nitrate was determined with the Saville-Griess assay adapted for microtiter plates. A standard curve was prepared with serial dilutions (0–50 µM) of a freshly prepared sodium nitrite (NaNO_2_) stock solution (100 mM). Cells were homogenized in 1× PBS and, after centrifugation, 200 µl of the supernatant were added to a well of a 96-well plate. Fifty microliters of sulfanilamide solution (4 mg/ml in 1 N HCl) were added to standards and samples. After 2-min incubation, 50 μl of N-(naphtyl)-ethylenediamine dihydrochloride solution (6 mg/ml in H_2_O) were added, followed by incubation for 5 min at room temperature and measuring absorbance at 540 nm with a Spectra Fluor Plus® instrument and XFluor® software (Tecan, Crailsheim).

### Analysis of Lipid Signaling Molecules

Bioactive lipids including sphingolipids and ceramides, lysophosphatidic acids, and endocannabinoids were analyzed by liquid chromatography-electrospray ionization-tandem mass spectrometry (LC-ESI-MS/MS) as described in detail in the supplementary material of [85]. All analytical methods were optimized based on previous methods [7, 32, 85].

In brief, the analytes were extracted using liquid–liquid-extraction. Sample volumes were 10 µl for sphingolipids, 50 µl each for LPA and 100 μl for endocannabinoids. The quantification of all analytes was performed using a hybrid triple quadrupole-ion trap mass spectrometer QTRAP 5500 or 6500 + (Sciex, Darmstadt, Germany) equipped with a Turbo-V-source operating in positive ESI mode for sphingolipids and endocannabinoids and in negative ESI mode for LPA.

Sphingolipids were separated using an Agilent 1200 HPLC system equipped with a Zorbax C18 Eclipse Plus UHPLC column (50 × 2.1 mm, 1.8 μm, Agilent technologies, Waldbronn, Germany) and the analysis of LPA was done on the same HPLC system using a Luna C18 column (50 × 2 mm, 5 μm, Phenomenex, Aschaffenburg, Germany). Analysis of the endocannabinoids was done using an Agilent 1290 Infinity I UHPLC system equipped with an Acquity UPLC BEH C18 UPLC column (100 × 2.1 mm, 1.7 μm, Waters, Eschborn, Germany).

Quality control samples of three different concentration levels (low, middle, high) were run as initial and final samples of each run. For all analytes, the concentrations of the calibration standards, quality controls, and samples were evaluated by Analyst software 1.6.3 and MultiQuant software 3.0.2 (Sciex) using the internal standard method (isotope-dilution mass spectrometry) as described in [86]. Variations in accuracy were less than 15% for at least 67% of all QC samples. For the lower limit of quantification, a variation of 20% was accepted.

### Untargeted Lipidomic Analyses

Twenty microliter plasma or 40 µl lymph nodes homogenates (homogenated in 0.025 µg/ml water/ethanol 1:3 (v/v)) were extracted using methyl-tert-butyl-ether [87]. The organic phase was split into two aliquots, one for analysis in negative ion mode and the other in positive ion mode. After drying under a nitrogen stream at 45 °C, the aliquots were reconstituted in 120 µl methanol or stored at − 40 °C until analysis. LC-MS analysis was performed on a Nexera X2 system (Shimadzu Corporation, Kyoto, Japan) coupled to a TripleTOF 6600 (Sciex). The chromatographic separation was done on a Zorbax RRHD Eclipse Plus C8 1.8 µm 50 × 2.1 mm ID column (Agilent, Waldbronn, Germany) with a SecurityGuard Ultra C8 pre-column (Phenomenex, Aschaffenburg, Germany), using a binary gradient with 40 °C column temperature and a flow rate of 0.3 ml/min. For the positive mode, the mobile phase A consisted of 10 mM ammonium formate and 0.1% formic acid in water and mobile phase B of 0.1% formic acid in acetonitrile/isopropanol 2:3 (v/v). For measurement in negative mode, 1 mM ammonium formate and 0.1% formic acid in water was used as for mobile phase A. The MS analysis encompasses a TOF MS scan from 100 to 1000 m/z with six data-dependent acquisitions per cycle and a mass range of 50–1000 m/z. The identification of the lipid species was based on the exact mass (± 5 ppm), the isotope ratio and the comparison of the MS/MS spectra with the reference spectra according to LIPID MAPS (http://www.lipidmaps.org), METLIN (http://metlin.scripps.edu), or the Human Metabolome Database (HMDB, version 4.0).

To reduce the impact of small variations in instrument sensitivity during the measurements, all samples were randomized prior to analysis. Quality control samples were injected at the start and at the end of a run and after every 10th sample to verify system stability. Data evaluation was done with Analyst TF 1.7 and MultiQuant software 3.0, and peak areas were normalized to the quality control samples using median peak ratios by MarkerView software 1.2 (all Sciex).

### Microarray and RNAseq Data Analysis

Microarray data of GEO dataset GSE60847 (own previous data) were reanalyzed and searched for genes involved in lipid metabolisms, regulation, or function. Normalized data were analyzed with ArrayStar, which uses general linear models to assess differential expression. Data were log2 transformed, scored according to “fold-regulation,” *P* value and abundance, and top scored genes were then clustered using Euclidean distance metrics. Valid genes (above intensity threshold) are displayed as Volcano plots, showing the log2 difference, i.e., fold change, positive for upregulated genes and negative for downregulated genes, versus the –log10 of the *t*-test *P* value. The *P* value was set at 0.05 and adjusted according to Benjamini–Hochberg. Genes were text-filtered based on gene descriptions and GO ontology terms to find lipid regulating and metabolizing genes and genes involved in BH4 pathways (synthesis, recycling, and coenzyme functions).

RNAseq data of GSE95401 were analyzed accordingly, starting with the raw per-gene count data table provided by the authors of GSE95401. Genes were filtered as above and normalized as fold changes versus the mean of the controls, which were pooled from sub-experiments, resulting in *n* = 16 controls, and *n* = 9 for each disease model, namely epilepsy, EAE, stroke, and traumatic brain injury (TBI). “Lipid-genes” were searched as explained above, and data of EAE mice were compared to the control group by 2-way analysis of variance (ANOVA) and adjustment of *P* by controlling the false discovery rate (FDR) according to the two-step method of Benjamin, Krieger, and Yekutieli.

### Statistics

Group data are presented as mean ± SD or median ± interquartile range (IQR) for non-parametric data as specified in the respective figure legends. Data were analyzed with SPSS 25 and GraphPad Prism 8.3 and Origin Pro 2020. Data were mostly normally distributed or log-normally distributed. For testing the null hypothesis that groups were identical, two groups were compared with 2-sided, unpaired Student’s *t* tests. The Mann–Whitney *U* test (2 groups) or Kruskal–Wallis (> 2 groups) were used as non-parametric alternatives in case of violations of *t* test requirements. Time course data or multifactorial data were submitted to 2-way ANOVA using, e.g., the factors “time” and “genotype.” In case of significant differences, groups were mutually compared at individual time points using post hoc *t* tests according to Dunnett, i.e., versus the control group, or according to Šidák. For time courses of non-parametric scores, the Friedmann test was used. Asterisks in figures show multiplicity-adjusted *P* values.

Multivariate analyses of multiple lipid classes were used to reduce the dimensionality. Because raw lipid concentrations of different classes differ by several orders of magnitude, lipids were normalized and are expressed as percentage of the 90% quantile. Canonical discriminant analysis (CanDisc) was employed to separate treatment groups and to assess the predictability of group membership. Partial least square (PLS) analysis was used if analytes exceeded the number of samples per group. Score plots and 95% confidence ellipses were created in OriginPro2020. Untargeted lipidomic data (normalized peak areas) were log2 transformed. Volcano plots were used to show the log2 difference (fold difference) versus the –log10 of the *t* test *P* value. Lipids of interest were further analyzed using 2-way ANOVAs for “lipid × treatment,” and subsequent *t* test for “treatment.” Gene regulations were considered significant at a FDR < 0.05.

## Supplementary Information

Below is the link to the electronic supplementary material.

Supplementary file1 (PDF 508 KB)

Supplementary file2 (PDF 1636 KB)

Supplementary file3 (PDF 465 KB)

Supplementary file4 (PDF 57 KB)

Supplementary file5 (PDF 534 KB)

Supplementary file6 (PDF 517 KB)

Supplementary file7 (PDF 1625 KB)

Supplementary file8 (PDF 544 KB)

Supplementary file9 (PDF 526 KB)

Supplementary file10 (PDF 552 KB)

Supplementary file11 (PDF 57 KB)

Supplementary file12 (PDF 526 KB)

Supplementary file13 (PDF 449 KB)

Supplementary file14 (PDF 467 KB)

Supplementary file15 (PDF 1565 KB)

Supplementary file16 (DOCX 5324 KB)

## Data Availability

Microarray datasets have been deposited previously and are available as GEO dataset with the accession number GSE60847 [39] and GSE95401 [41].

## Abbreviations

AGMO: Alkyglycerol monooxygenase
BBB: Blood–brain barrier
BH4: Tetrahydrobiopterin
EAE: Experimental autoimmune encephalomyelitis
DAHP: Diaminohydroxypyrimidine
GCH1: GTP cyclohydrolase 1
MS: Multiple sclerosis
NOS: Nitric oxide synthase
RRMS: Relapsing remitting MS
PPMS: Primary progressive MS
SPMS: Secondary progressive MS
PTPS: 6-Pyrovoyltetrahydropterin synthase
SPR: Sepiapterin reductase

## Declarations

### Required Author Forms

Disclosure forms provided by the authors are available with the online version of this article.
