# Supplementary material for: Sapropterin (BH4) Aggravates Autoimmune Encephalomyelitis in Mice
Source: Neurotherapeutics. 2021 Apr 12;18(3):1862–79. doi: 10.1007/s13311-021-01043-4 (PMC8609075; doi:10.1007/s13311-021-01043-4)
Supplement: Supplementary file 15 — Supplementary file15 (PDF 1565 KB) [file 13311_2021_1043_MOESM15_ESM.pdf]

Supplement to

Sapropterin (BH4) aggravates autoimmune encephalomyelitis in mice

Katja Schmitz[1], Sandra Trautmann[1], Lisa Hahnefeld[1], Caroline Fischer[1], Yannick Schreiber[1], Robert Brunkhorst[2], Ernst R. Werner[3], Katrin Watschinger[3], Sabine Wicker[4], Dominique Thomas[1], Gerd Geisslinger[1,5,6], Irmgard Tegeder[1]

Summary

Depletion of the enzyme cofactor, tetrahydrobiopterin (BH4) in T-cells was shown to prevent their proliferation upon receptor stimulation in models of allergic inflammation in mice suggesting that BH4 drives autoimmunity. Hence, the clinically available BH4 drug (sapropterin) might increase the risk of autoimmune diseases. The present study assessed the implications for multiple sclerosis (MS) as an exemplary CNS autoimmune disease. Plasma levels of biopterin were persistently low in MS patients and tended to be lower with high Expanded Disability Status Scale (EDSS). Instead, the bypass product, neopterin was increased. The deregulation suggested that BH4 replenishment might further drive the immune response or beneficially restore the BH4 balances. To answer this question, mice were treated with sapropterin in immunization-evoked autoimmune encephalomyelitis (EAE), a model of multiple sclerosis. Sapropterin-treated mice had higher EAE disease scores associated with higher numbers of T-cells infiltrating the spinal cord, but normal T-cell subpopulations in spleen and blood. Mechanistically, sapropterin-treatment was associated with increased plasma levels of long-chain ceramides and low levels of the poly-unsaturated fatty acid, linolenic acid (FA18:3). These lipid changes are known to contribute to disruptions of the blood brain barrier in EAE mice. Indeed, RNA data analyses revealed upregulations of genes involved in ceramide synthesis in brain endothelial cells of EAE mice (LASS6/CERS6, LASS3/CERS3, UGCG, ELOVL6 and -4). The results support the view that BH4 fortifies autoimmune CNS disease, mechanistically involving lipid deregulations that are known to contribute to the EAE pathology.

Suppl. Table 1A

Demographic data of MS patients and Healthy Controls with serum samples

	Patients with Multiple Sclerosis, serum samples								Healthy Controls (HC)	
	First course		RRMS stable		RRMS acute relapse		SPMS or PPMS			
Gender	female	male	female	male	female	male	female	male	female	male
Age (mean)	31.64	38.23	34.3	35.48	37.16	33.85	49.25	43.57	24.9	25.03
Age (SD)	9.14	7.52	8.87	9.41	10.91	7.03	10.96	18.08	6.08	6.13
Disease years (mean)	0.88	0.1	7.41	7.55	6.68	2.08	1.05	5.67		
Disease years (SD)	2.87	0.04	5.86	6.1	5.27	1.77	1.34	4.67		
	Number of patients		Number of patients		Number of patients		Number of patients		Number of HC	
No drug	11	6	6	3	10	1	1	1	183	118
Interferon	0	0	11	6	5	2	1	0		
Fingolimod	0	0	5	3	1	0	0	1		
Natalizumab	0	0	15	4	0	0	0	0		
Other (e.g. glatiramer, fumarate, AZA)	1	2	2	0	2	1	0	1		
**Total**	**12**	**8**	**39**	**16**	**18**	**4**	**2**	**3**	**183**	**118**
**Sum**								**102**		**301**

Suppl. Table 1B

Demographic data of MS patients of cohort-2 for individual time courses

Days	Day of diagnosis set to 0	Therapy	Clinical	Pat#	Sample	Patient	Biopterin (ng/ml)	Neopterin (ng/ml)
426	0	Steroid	Relapse	#1	1	A	1.067	4.949
576	150	NTZ	preRelapse	#1	2	A	0.898	3.952
943	517	NTZ	Interval	#1	3	A	1.159	5.015
224	0	Steroid	Relapse	#2	4	B	1.238	3.966
404	180	Steroid	Relapse	#2	5	B	1.359	4.418
610	386	Steroid	postRelapse	#2	6	B	1.111	3.434
1497	1273	n.a.	Interval	#2	7	B	1.214	3.227
518	0	Fingolimod	preRelapse	#3	8	C	1.436	5.211
562	44	n.a.	Relapse	#3	9	C	2.181	6.497
661	143	Fingolimod	Interval	#3	10	C	2.261	5.948
749	231	Fingolimod	Interval	#3	11	C	3.545	5.982
254	0	NTZ	preRelapse	#4	12	D	1.198	5.198
673	419	n.a.	Relapse	#4	13	D	1.77	5.661
742	488	NTZ	Interval	#4	14	D	1.451	5.487
937	683	NTZ	Interval	#4	15	D	1.305	4.688
199	0	noTreat	preRelapse	#5	16	E	1.035	4.922
199	0	noTreat	Relapse	#5				
765	566	Fingolimod	Relapse	#5	17	E	2.127	4.952
765	566	Fingolimod	Relapse	#5	18	E	1.293	4.216
1095	896	NTZ	Interval	#5				
1187	988	NTZ	Interval	#5	19	E	1.183	3.863
1213	1014	NTZ	Interval	#5	20	E	1.357	4.392

1213	1014	NTZ	Interval	#5				
0	0	NTZ	preRelapse	#6	21	F	1.501	3.432
112	112	n.a.	Relapse	#6	22	F	1.353	3.305
191	191	NTZ	Interval	#6	23	F	1.636	3.672
247	247	NTZ	Interval	#6	24	F	1.749	4.291
213	0	before	Relapse	#7	25	G	1.296	9.426
735	522	before	Interval	#7	26	G	1.604	13.299
915	702	NTZ	Interval	#7	27	G	1.337	2.53
1146	933	NTZ	Interval	#7	28	G	1.473	2.976
1185	0	NTZ	Interval	#8	29	H	0.905	7.411
1254	69	n.a.	Relapse	#8	30	H	1.168	4.149
1405	220	NTZ	Interval	#8	31	H	1.04	7.089
1467	282	NTZ	Interval	#8	32	H	0.92	5.817
529	0	NTZ	preRelapse	#9	33	I	1.432	4.168
638	109	Steroid	Relapse	#9	34	I	2.061	3.946
1124	595	RTX	postRelapse	#9	35	I	1.718	7.321
1191	0	NTZ	preRelapse	#10	36	J	1.74	4.9
1383	192	n.a.	Relapse	#10	37	J	1.277	3.67
1405	214	NTZ	postRelapse	#10	38	J	1.233	4.554
1472	281	Steroid	Relapse	#10	39	J	1.372	4.225
1490	299	Steroid	Relapse	#10	40	J	1.187	5.907
1566	375	NTZ	Interval	#10	41	J	1.13	3.89
184	0	noTreat	Interval	#11	42	K	1.923	2.868
246	62	Fingolimod	Interval	#11	43	K	1.427	4.077
158	0	noTreat	Relapse	#12	44	L	0.917	10.296
350	192	Fingolimod	Interval	#12	45	L	1.104	6.375
1185	1027	Fingolimod	Interval	#12	46	L	1.183	4.562
1247	0	NTZ	preRelapse	#13	47	M	0.934	4.047
1383	136	n.a.	Relapse	#13	48	M	1.033	4.733
1496	249	NTZ	Interval	#13	49	M	0.949	3.39
494	0	noTreat	Relapse	#14	50	N	1.233	5.79
499	5	Fingolimod	Interval	#14	51	N	2.357	5.769
1214	720	Fingolimod	Interval	#14	52	N	1.673	3.464

Suppl. Table 2

Antibodies

Antibody	*Type*	*Host*	*Conjugate*	*Company*	*Product #*
CD11b	mab	rat	unconjugated	BioRad	MCA711
CD11b	mab	rat	APC	Miltenyi Biotec	130-091-241
CD11b	mab	rat	eFluor 450	eBioscience	48-0112-80
CD11c	mab	hamster	eFluor 450	eBioscience	48-0114-80
CD19	mab	rat	PE	eBioscience	12-0193-82
CD206	mab	mouse	FITC	Biozol	BZL 20766
CD25	mab	rat	PE-Cy7	eBioscience	25-0251-81
CD3e	mab	hamster	unconjugated	BD	550275
CD3	mab	rat	APC-Cy7	BioLegend	100330
CD36	mab	rat	PE	eBioscience	12-0361-81
CD4	mab	rat	eFluor 500	BD	560782
CD45	mab	rat	eFluor 450	eBioscience	48-0451-82
CD8	mab	rat	FITC	eBioscience	11-0083-81
F4/80	mab	rat	unconjugated	eBioscience	14-4801-82
F4/80	mab	rat	PE-Cy7	eBioscience	25-4801-82
Iba1	pab	rabbit	unconjugated	WAKO	019-19741
IFN-γ	mab	rat	AF488	eBioscience	53-7311-82
MHC-II	mab	rat	PerCP-eFluor710	eBioscience	46-5320-80
Ly6C	mab	rat	PerCP-Cy5.5	eBioscience	45-5932-80
Ly6G	mab	rat	APC-Cy7	BD	560600
NeuN	mab	mouse	unconjugated	Merck Millipore	MAB377

Supplementary figures and legends

Suppl. Figure 1

Tetrahydrobiopterin synthesis and coenzyme functions

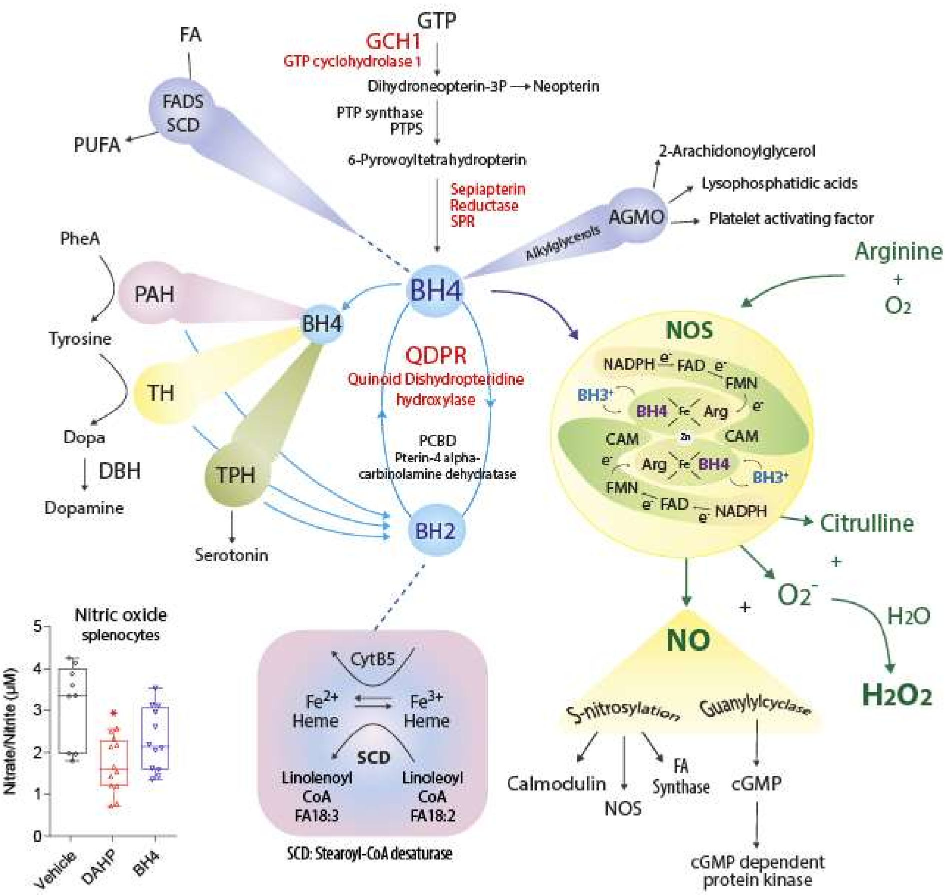

Suppl. Figure 1:

Tetrahydrobiopterin synthesis and coenzyme functions and effects of GCH1 inhibition in splenocytes

The graph shows the biosynthesis of tetrahydrobiopterin (BH) and its coenzyme functions and also illustrates putative effects on iron dependent fatty acid metabolism. The box/scatter plot shows nitrite/nitrate levels obtained in the Griess assay in primary splenocytes, which were restimulated with 25 ng/ml IFNγ. The spleens were obtained from vehicle, DAHP or sapropterin (BH4) treated SJL/J-EAE mice 22 days after immunization. The box represents the interquartile range, whiskers show minimum to maximum, the line is the median. Data were compared with one-way ANOVA and subsequent posthoc t-test with adjustment of alpha according to Šidák (* $P < 0.05$).

Suppl. Figure 2

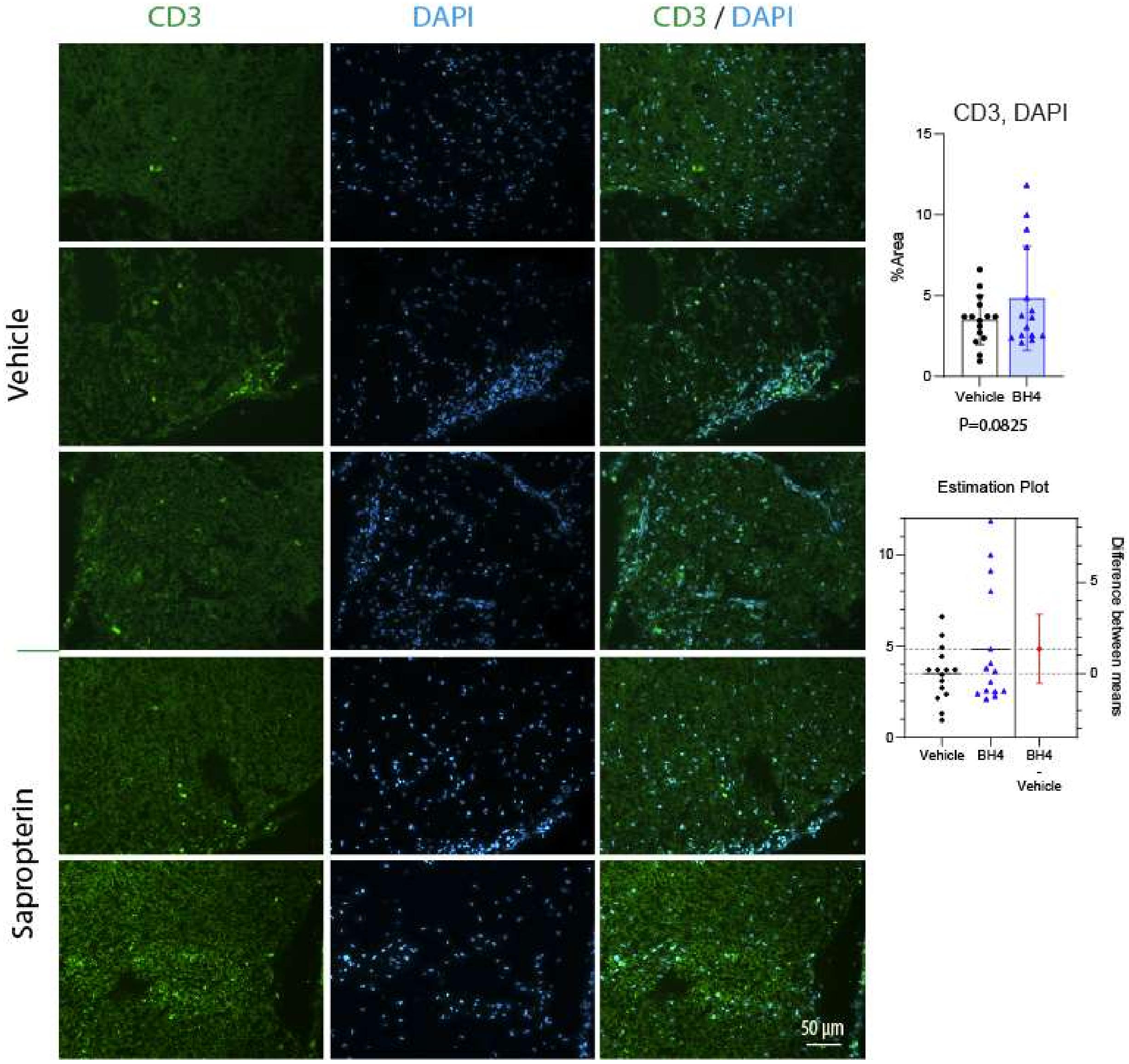

Suppl. Figure 2

Immunofluorescence of CD3 in spinal cord sections of EAE mice treated with sapropterin or vehicle

Cryosections of the lumbar spinal cord were subjected to immunofluorescence analysis using anti-CD3 antibody to reveal T-cells. CD3 (green) was counterstained with DAPI to reveal nuclei. The right panel shows the merged images. CD3 immunofluorescence signals were quantified in 4-5 sections per mouse of n = 3 mice per treatment group in FIJI ImageJ using the Particle Counter after background subtraction and default threshold settings with minor adjustment. The relative CD3 immunoreactive area (relative to the total image size, equal for all analyses) was used for group comparisons. Data were compared by Student's t-test.

Suppl. Figure 3

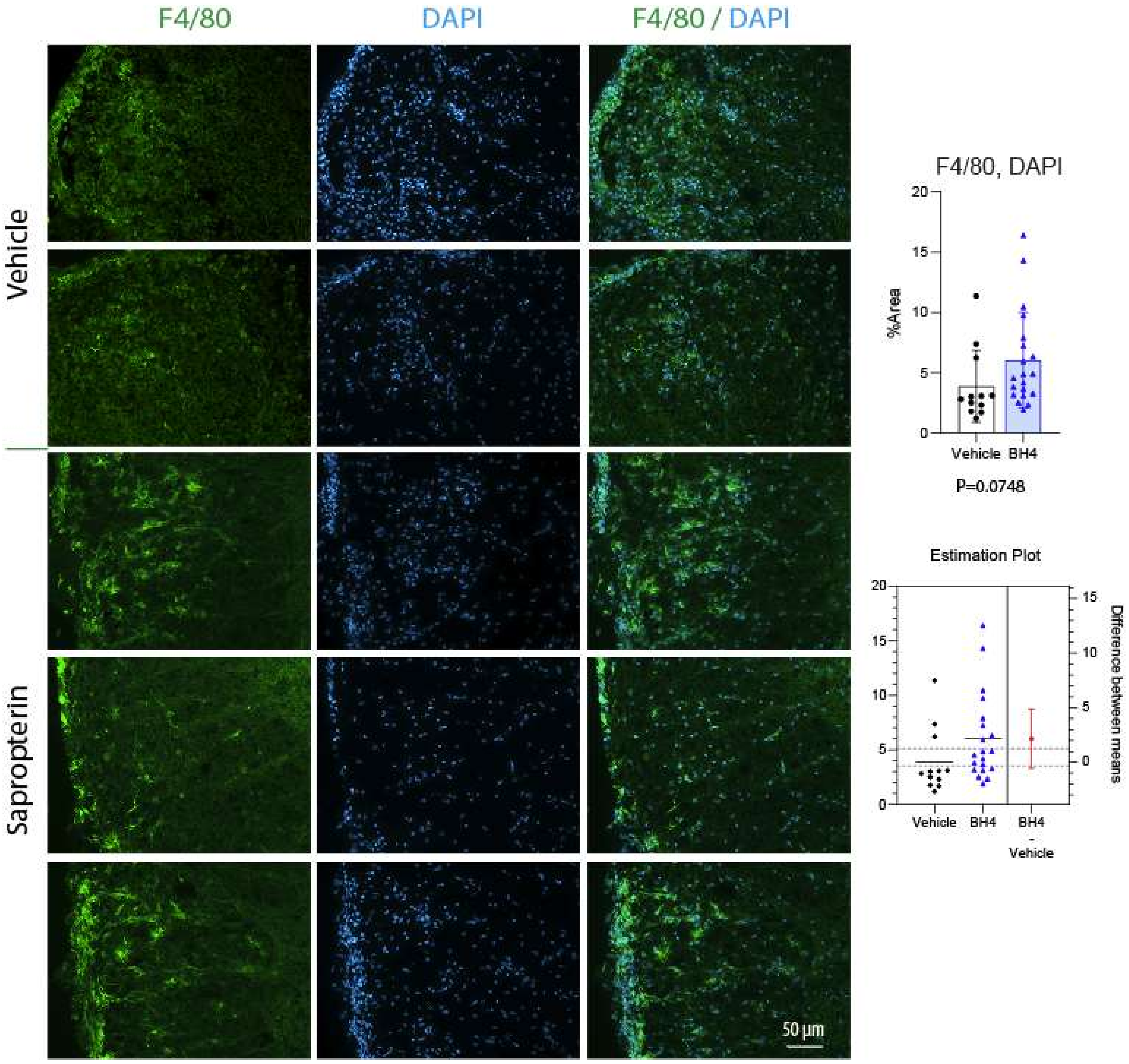

Suppl. Figure 3

Immunofluorescence of F4/80 in spinal cord sections of EAE mice treated with sapropterin or vehicle

Cryosections of the lumbar spinal cord were subjected to immunofluorescence analysis using anti-F4/80 antibody to reveal myeloid derived cells. F4/80 (green) was counterstained with DAPI to reveal nuclei. The right panel shows the merged images. The quantification was done as in Suppl. Fig. 2. Data are from 4-5 sections per mouse of 3 mice per group.

Suppl. Figure 4

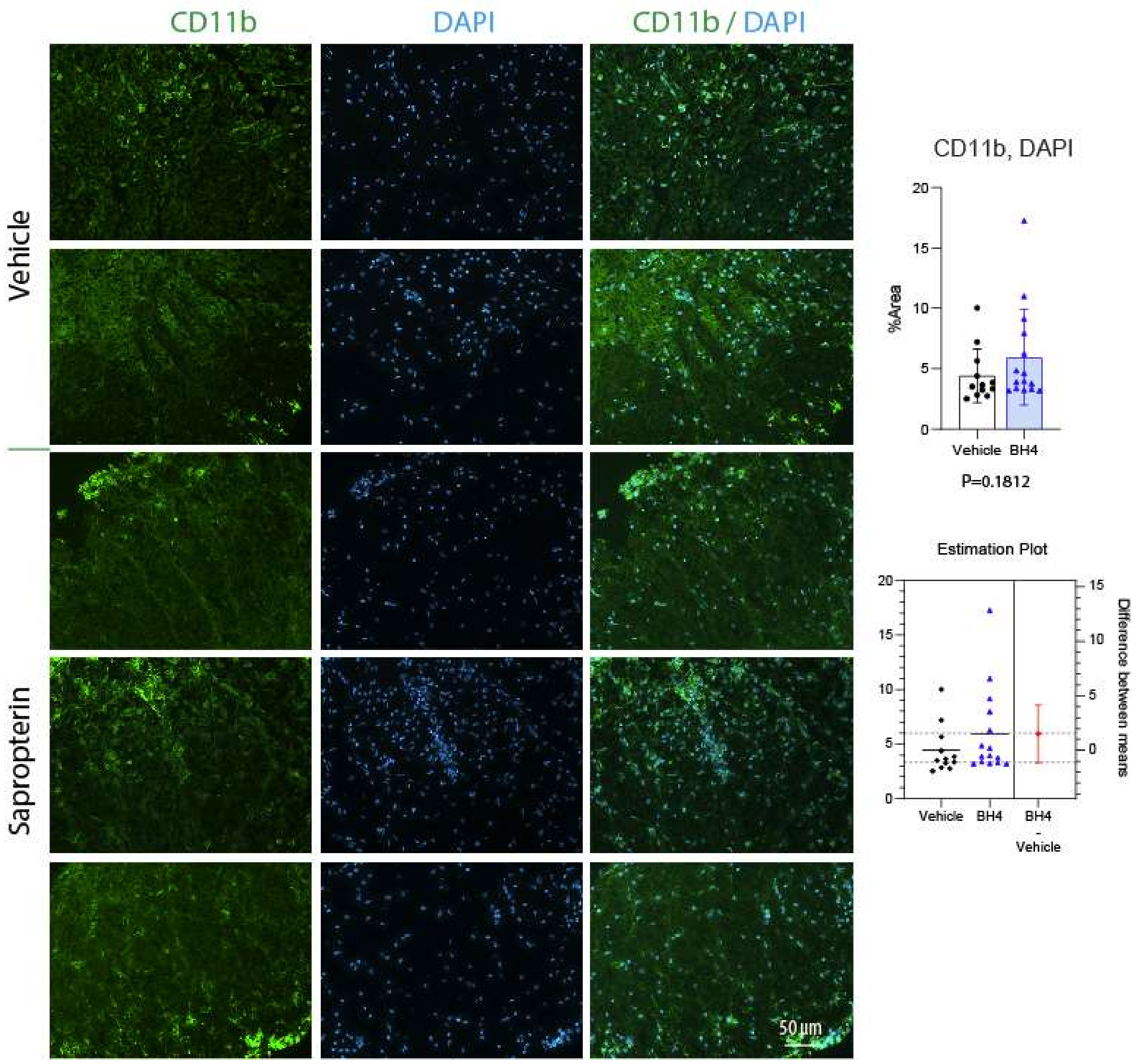

Suppl. Figure 4

Immunofluorescence of CD11b in spinal cord sections of EAE mice treated with sapropterin or vehicle

Cryosections of the lumbar spinal cord were subjected to immunofluorescence analysis using anti-CD11b antibody to reveal complement-3 receptor positive myeloid derived cells. Microglia have high CD11b, peripheral macrophages have moderate CD11b expression. CD11b (green) was counterstained with DAPI to reveal nuclei. The right panel shows the merged images. The quantification was done as in Suppl. Fig. 2. Data are (mean ± SD) are from 4-5 sections per mouse of 3 mice per group.

Suppl. Figure 5

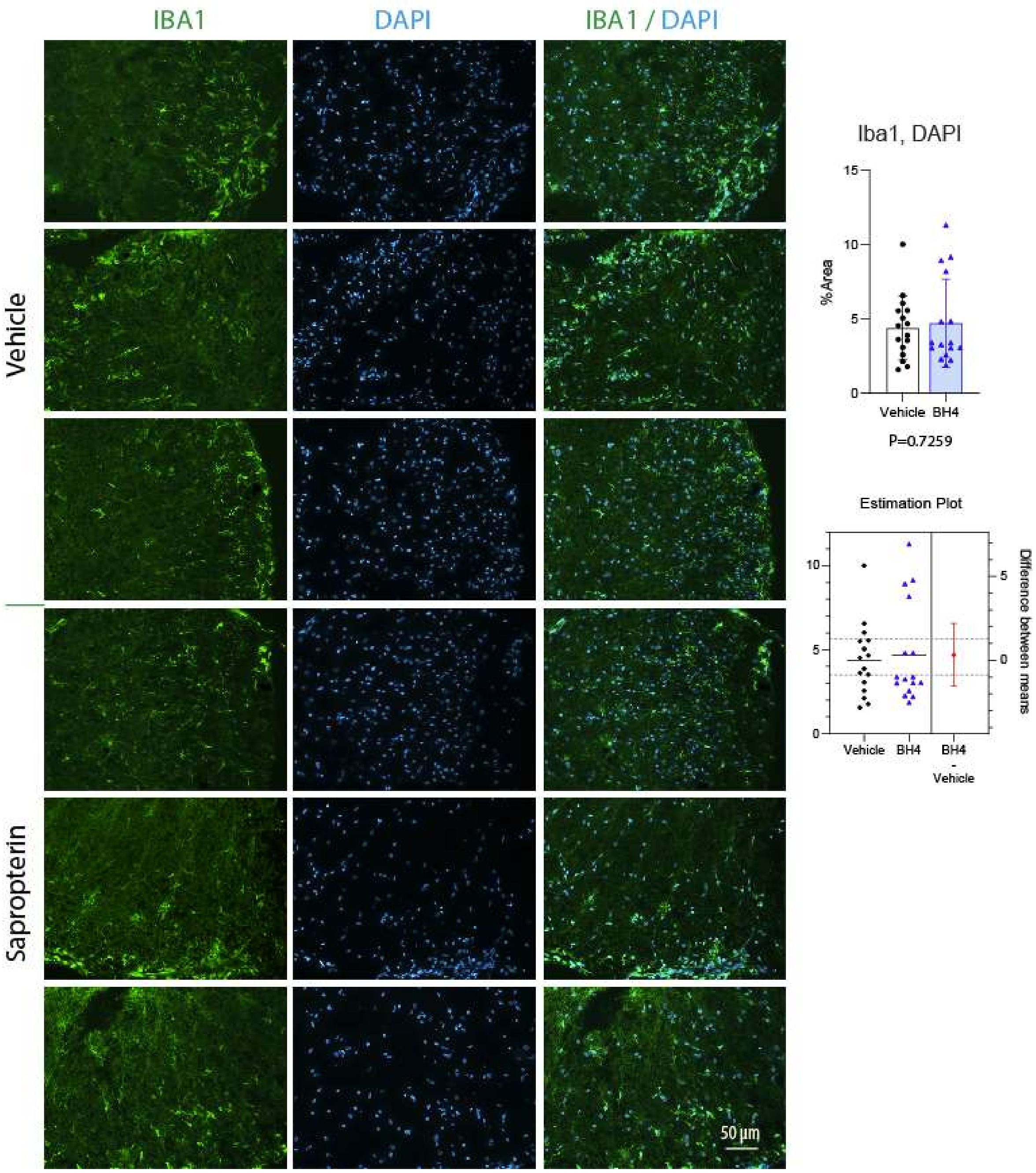

Suppl. Figure 5

Immunofluorescence of Iba1 in spinal cord sections of EAE mice treated with sapropterin or vehicle

Cryosections of the lumbar spinal cord were subjected to immunofluorescence analysis using anti-Iba1 antibody to reveal allograft inflammatory factor 1 positive myeloid derived cells. Microglia and infiltrating macrophages express Iba1. Iba1 (green) was counterstained with DAPI to reveal nuclei. The right panel shows the merged images. The right panel shows the merged images. The quantification was done as in Suppl. Fig. 2. Data are (mean ± SD) are from 4-5 sections per mouse of 3 mice per group.

Suppl. Figure 6

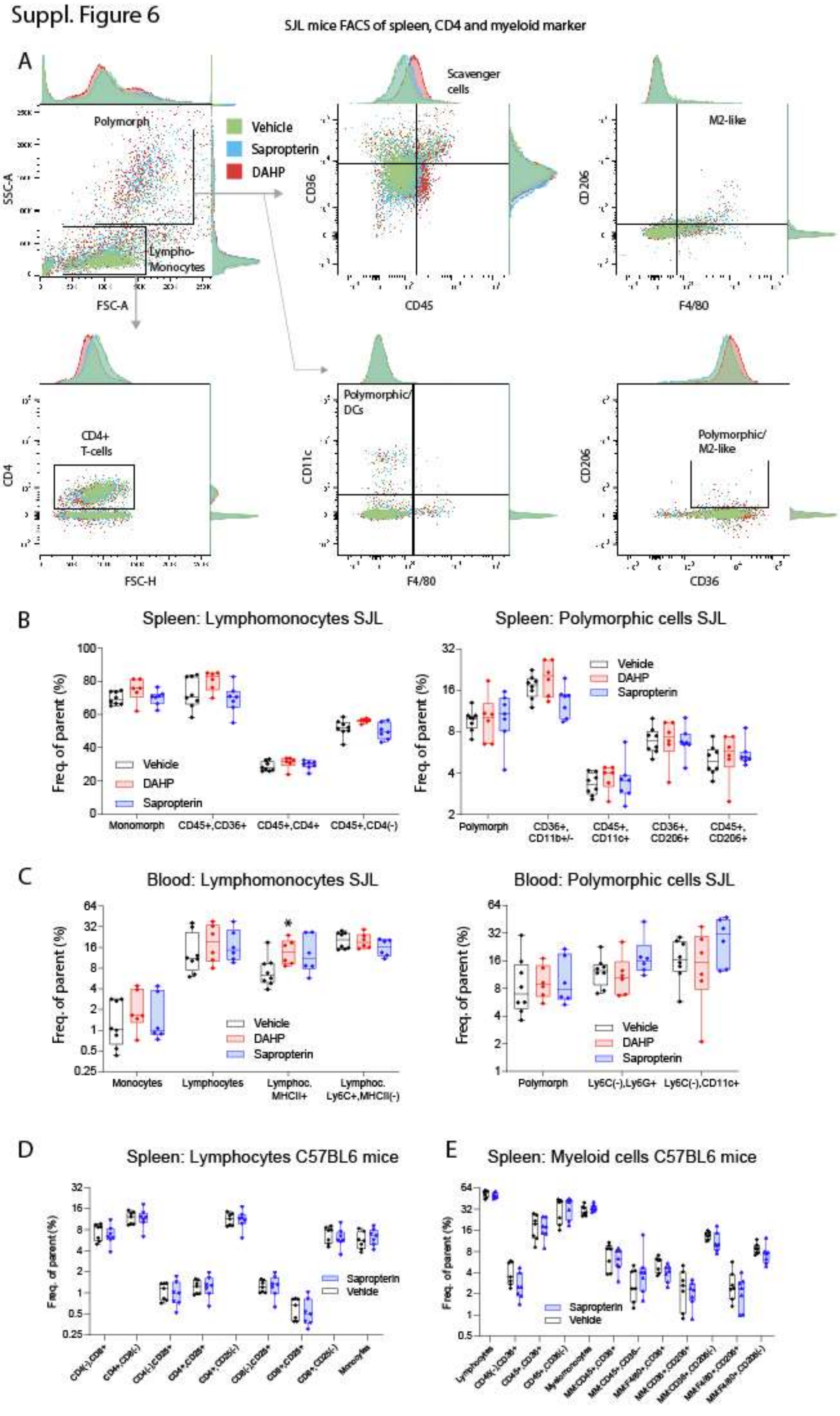

Suppl. Figure 6:

FACS analyses of lymphocytes and myeloid cells in spleen and blood in EAE mice treated with sapropterin or vehicle

A: Exemplary results of the FACS analysis of the splenocytes from vehicle, DAHP or sapropterin (BH4) treated SJL/J-EAE mice. The tissue was dissected 22 days after immunization. 100000 cells were counted of each 6-8 mice. Exemplary dot plots show splenic cells gated according to forward scatter (FSC) versus sideward scatter (SSC). The framed areas show lympho-/monocytes and a gate of polymorphic cells. The polymorphic cells were further analyzed for CD45/CD36 (scavenger cells) and F4-80/CD206 (M2-like macrophages). Lympho-/monocytes were further analyzed for CD4 (T-cells), F4-80/CD11c (dendritic cells) and CD36/CD206 (M2-like).

B: Results of FACS analysis shown in A. The data show the frequency (percentage of the parent population). Data were compared with two-way ANOVA "population X treatment" and subsequent posthoc analysis for treatment using an adjustment of alpha according to Šidák (n = 6-8 per group).

C: FACS analyses of circulating blood cells from vehicle, DAHP or sapropterin (BH4) treated SJL/J-EAE mice. Statistics as in B.

D: FACS analysis of splenocytes for lymphocytic subpopulations from vehicle or sapropterin (BH4) treated C57Bl6/J-EAE mice. The tissue was dissected 19 days after immunization. 100000 cells were counted of each 7 mice. There was no difference between groups.

Suppl. Fig. 7

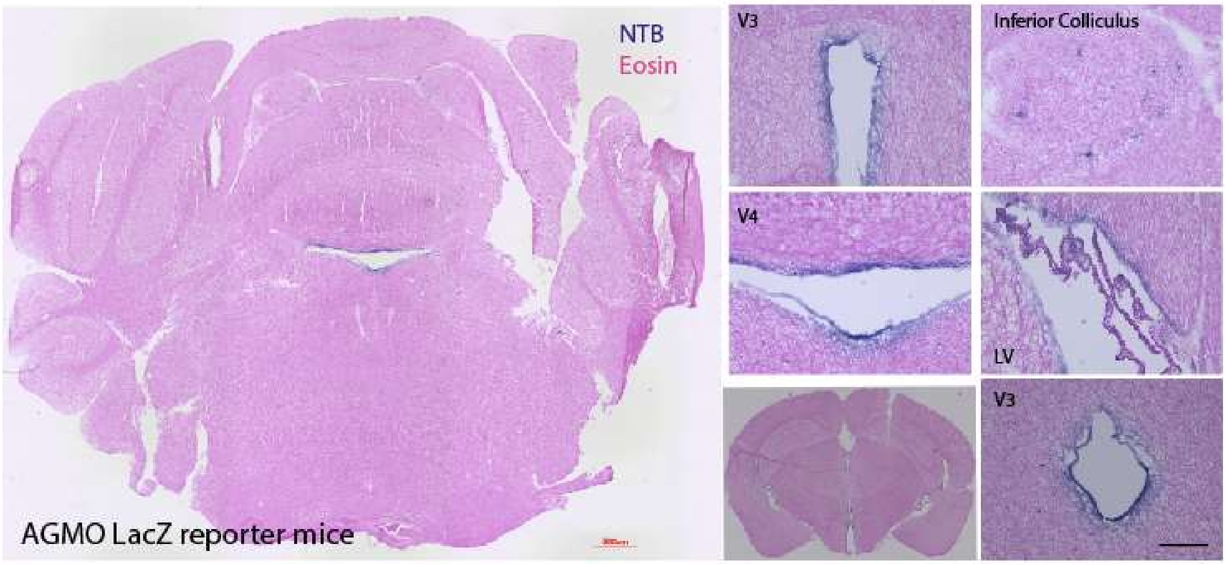

Suppl. Figure7

Exemplary images of nitrotetrazolium blue (NTB) staining of the brain of adult AGMO LacZ reporter mice. AGMO expressing cells are blue. Sections were counterstained with eosin (red). Scale bar 500 µm (overview) or 100 µm. V, Ventricle.

Suppl. Fig. 8

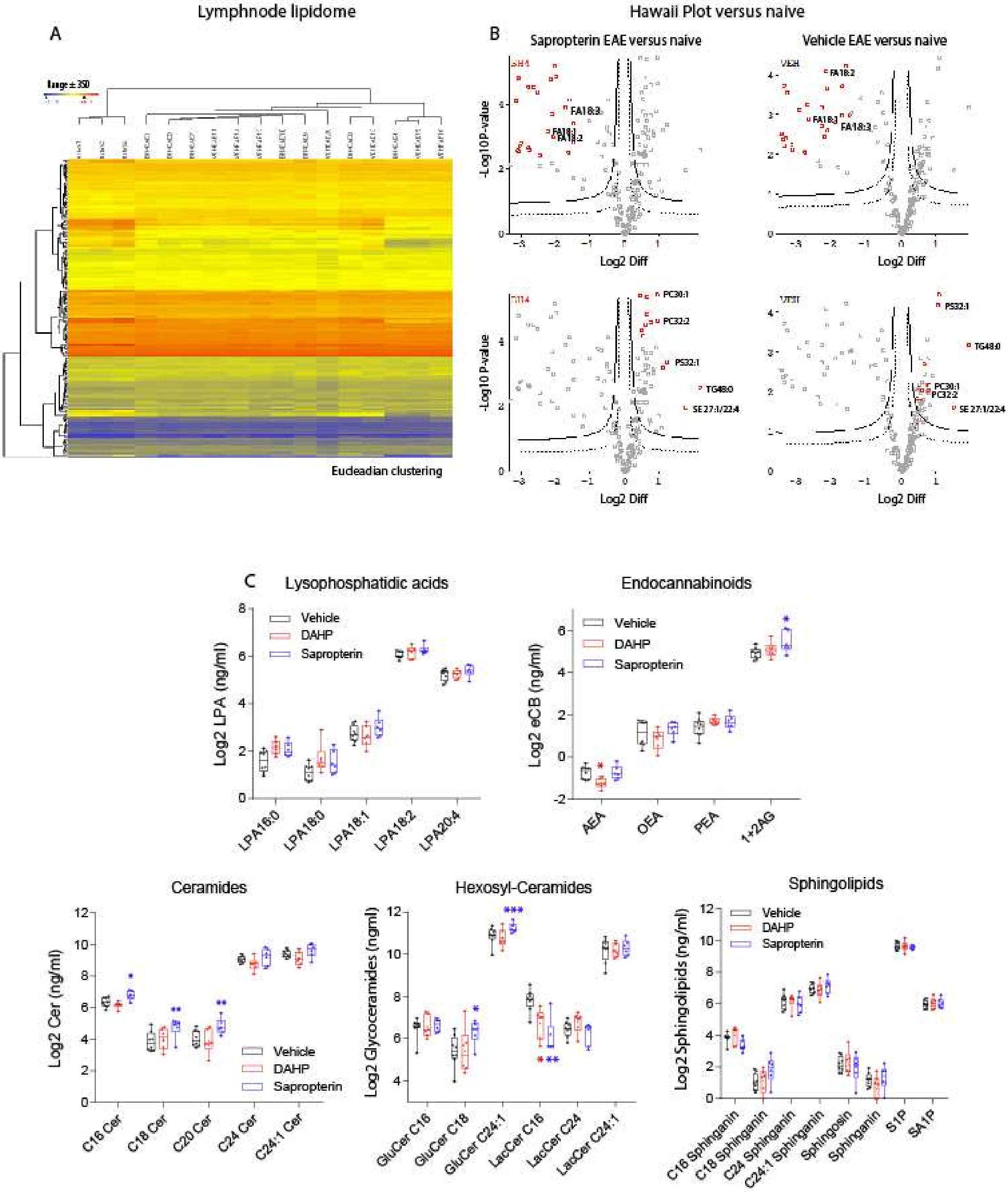
Lymphnode lipidome
A
Eucleadian clustering
Hawaii Plot versus naive
B
Sapropterin EAE versus naive
Vehicle EAE versus naive
-Log10 P-value
Log2 Diff
FA18:3
FA18:1
FA18:2
PC30:1
PC32:2
PS32:1
TG48:0
SE 27:1/22:4
C
Lysophosphatidic acids
Vehicle
DAHP
Sapropterin
Log2 LPA (ng/ml)
LPA16:0
LPA18:0
LPA18:1
LPA18:2
LPA20:4
Endocannabinoids
Log2 eCB (ng/ml)
AEA
OEA
PEA
1+2AG
Ceramides
Log2 Cer (ng/ml)
C16 Cer
C18 Cer
C20 Cer
C24 Cer
C24:1 Cer
Hexosyl-Ceramides
Log2 Glycoceramides (ngml)
GluCer C16
GluCer C18
GluCer C24:1
LacCer C16
LacCer C24
LacCer C24:1
Sphingolipids
Log2 Sphingolipids (ng/ml)
C16 Sphinganin
C18 Sphinganin
C24 Sphinganin
C24:1 Sphinganin
Sphingosin
Sphinganin
S1P
SA1P

Suppl. Figure 8

A: Heatmap of the lymph node lipidome of naïve C57BL6 and EAE mice treated with sapropterin or vehicle. Treatment was started at the day of immunization and tissue was dissected 19 days after immunization. Lipid concentrations are color-coded (red high, blue low, color bar in the top right corner). Lipids and mice were clustered using Euclidean distance metrics. Dendrograms show lipid (rows) and mouse clusters (columns). Naïve mice were separated from EAE mice but sapropterin and vehicle treated mice were similar.

B: Hawaii plots show the lymph node lipidome of sapropterin treated and vehicle treated of EAE mice, versus naïve mice. The upper row highlights reduced lipids in EAE (red), the bottom row highlights increased lipids. Each plot is a Volcano plot that shows the Log2 difference (= fold difference; X-axis) versus the negative logarithm of the t-test P value (Y-axis). Some candidates are labeled with the standard lipid name.

C: Box/scatter plots of plasma lipids of various classes in vehicle, DAHP or sapropterin (BH4) treated SJL/J-EAE mice. The box represents the interquartile range, whiskers show minimum to maximum, the line is the median. Each scatter is a mouse. Data were compared with two-way ANOVA for "lipid X treatment" and subsequent posthoc analysis for treatment using an adjustment of alpha according to Šidák (n = 6-8 per group, * P < 0.05, ** P < 0.001).

References

[1] Munji RN, Soung AL, Weiner GA, et al. Profiling the mouse brain endothelial transcriptome in health and disease models reveals a core blood-brain barrier dysfunction module. Nat. Neurosci. 2019;22(11): 1892-1902.
